# Improving the Sensory Quality of Black Tea by Blending Varieties During Processing

**DOI:** 10.3390/foods14060941

**Published:** 2025-03-10

**Authors:** Wenxue Chen, Jiezhong Zan, Linfeng Yan, Haibo Yuan, Peiqiang Wang, Yongwen Jiang, Hongkai Zhu

**Affiliations:** 1 College of Horticulture, Qingdao Agricultural University, Qingdao 266109, China; 2 Key Laboratory of Tea Biology and Resource Utilization, Ministry of Agriculture Tea Research Institute, China Academy of Agricultural Sciences, Hangzhou 310008, China192168092@tricaas.com (H.Y.); jiangyw@tricaas.com (Y.J.); 3 Sichuan Tea Industry Group Co., Ltd., Yibin 644000, China

**Keywords:** fresh tea leaves, new process, oolong varieties, tea catechins, volatile components

## Abstract

Tea blending technology is based on finished tea. Blending fresh leaves during processing has not been proposed and investigated anywhere. This study investigates the impact of blending fresh leaves from different varieties on the flavor quality of black tea. The main taste components, including catechins, theaflavins, and free amino acids, were analyzed using HPLC, while the volatile components were analyzed using GC-MS. The results show that adding fresh *Jinguanyin* or *Jinxuan* leaves to *Fudingdabai* can regulate the ratio of esterified to non-esterified catechins, increase the content of theaflavins and amino acids, and positively impact the strength and freshness of the black tea. The sensory evaluation results show that the taste scores of FJG (black tea made from the blend of fresh *Fudingdabai* and *Jinguanyin* tea leaves) (92.14 ± 0.41 b) and FJX (black tea made from the blend of fresh *Fudingdabai* and *Jinxuan* tea leaves) (93.80 ± 0.19 a) are significantly higher than those of *Fudingdabai* (90.05 ± 0.31 d), *Jinguanyin* (86.10 ± 0.45 e), and *Jinxuan* (91.03 ± 0.26 c). Furthermore, adding fresh *Jinguanyin* or *Jinxuan* leaves to *Fudingdabai* can also enhance the floral compounds in the black tea, specifically phenylacetaldehyde, linalool, benzyl alcohol, and oxidized linalool (linalool-type pyran), which make important contributions to the floral aroma of the black tea. Conclusions: Blending fresh leaves for processing can enhance the sensory quality of black tea. This work proposes new insights and methods to enhance black tea sensory quality via the blending of fresh tea leaves with different varieties during processing.

## 1. Introduction

Black tea is widely consumed around the world due to its health benefits and rich flavors [1,2]. Several factors influence the quality of black tea, including the variety of the tea plant, the ecological environment in which it is grown, and the processing techniques used [3,4]. Traditional processing methods for black tea include withering, rolling, fermenting, and drying [5]. Producers often adjust these processing parameters to enhance the quality of black tea, but the change in flavor characteristics is limited. For example, *Fudingdabai* is currently the most widely planted tea variety for producing black tea [6]. The black tea made from it has a relatively balanced flavor and aroma, but it is difficult to create a distinctive floral-scented black tea from it [7].

The unique flavor characteristics of black tea are primarily determined by the tea variety, which is influenced by the varying chemical components present in different varieties. For example, a tea variety with a high amino acid content tends to have a pronounced umami and fresh taste, while varieties rich in catechins can produce a more mellow or “kokumi” taste, as well as bitterness and astringency [8]. The variety of *Camellia sinensis cv. Zhuye* is used to produce Keemum black tea and is known for its distinctive “Keemun aroma”, which features rose-like notes. In contrast, many oolong varieties that are cooked as black tea tend to present rich floral and fruity aromas. Therefore, to address the varying quality of the teas and meet diverse consumer preferences, different varieties of cooked tea products with varying characteristics are often blended before reaching the market [9].

The blending of tea has been defined as the proper mixing of more than one variety or grade of tea from various tea estates or regions, resulting in a unique or distinctive type of tea [10]. The blending process is typically manual, where the right proportions of ingredients are selected based on their appearance, aroma, and taste. This process creates a blended tea that is soothing, desirable, palatable, and price-competitive. However, this traditional blending method requires experienced technical personnel as well as a significant amount of time and effort. Additionally, the traditional blending method is influenced by subjective factors, which makes the quality of the tea not very stable. In recent years, new technologies have been used to improve blending methods, addressing the subjective influences on the results. Tie et al. [11] developed an intelligent approach using hierarchical spatial clustering-based algorithms for the blending process, which provided a universal, low-cost, and efficient blending program to aid in financial decision-making. Turgut, Küçüköner, and Karacabey [9] employed chemometric methods to develop an algorithm for determining blending proportions, accompanied by an easy-to-use graphical interface that simplifies and accelerates the tea blending process at a lower cost. Djeumou Fomeni [12] employed Monte Carlo simulation approaches to formulate a multi-objective model for the blending problem in the tea industry. However, the advancements in blending methods often represent mere modern adaptations of traditional manual techniques without fully reforming the complex and time-consuming tea-blending process involved. Research shows that the blending of fresh leaves from different varieties of raw materials can affect the levels of tea polyphenols, theaflavins, catechins, amino acids, and aroma [13]. If fresh tea leaves of different varieties are blended based on their individual strengths in aroma, taste, and other characteristics, then the blending can be incorporated throughout the entire production process, negating the need for post-production blending. This approach streamlines operations, making the process more convenient and cost-effective. This unique processing technique may result in higher quality products, providing tea producers with a differentiated market competitive advantage, thereby attracting more consumers, increasing market share, and generating higher profits. However, research on blending and processing fresh tea leaves has largely remained unexplored. Further investigation into the production of black tea using fresh leaf blending is of great significance for achieving efficient and high-quality black tea production.

To compare the effects of blending fresh leaves during processing on the quality of black tea, the experiment used *Fudingdabai*, a variety with relatively balanced sensory characteristics, and two oolong tea varieties, *Jinguanyin* and *Jinxuan*, known for their prominent floral aroma, as raw materials to prepare the black tea samples. The tea samples were analyzed using high-performance liquid chromatography (HPLC) and an automatic amino acid analyzer to detect the contents and components of catechins, theaflavins, and amino acids. Volatile compounds were determined by using solid-phase microextraction (SPME) with gas chromatography-mass spectrometry (GC-MS) to explore how blending treatments affected the aroma profiles. This work aims to evaluate how fresh leaf blending can improve the flavor and aroma quality of black tea compared to traditional blending methods.

## 2. Materials and Methods

### 2.1. Chemicals and Reagents

Gallocatechin (GC, ≥98%), epigallocatechin (EGC, ≥98%), catechin (C, ≥97%), epicatechin (EC, ≥98%), epigallocatechin gallate (EGCG, ≥99%), gallocatechin gallate (GCG, ≥98%), epicatechin gallate (ECG, ≥98%), catechin gallate (CG, ≥98%), caffeine (CAF, ≥99%), gallic acid (GA, ≥98%), theaflavin (TF1, ≥98%), theaflavin-3-gallate (TF2A, ≥98%), theaflavin-3′-gallate (TF2B, ≥98%), and theaflavin-3,3′-digallate (TF3, ≥98%) were purchased from Macklin Biochemical Co., Ltd. (Shanghai, China). Methanol (HPLC grade), isopropanol (HPLC grade), acetonitrile (HPLC grade), ethanol (HPLC grade), and acetic acid (HPLC grade) were purchased from Merck (Darmstadt, Germany). The mixture of amino acids standards and eluants were purchased from Sigma-Aldrich (Shanghai, China). The *n*-alkane mixtures of C7–C40 were purchased from Shanghai Yuanye Biotechnology Co., Ltd. (Shanghai, China). Ethyl decanoate (≥99%) was purchased from Aladdin Industrial Inc. (Shanghai, China). All other reagents were of analytical grade. Double-ionized water was produced from a purification system (IQ 7000, Merck-Millipore, St. Louis, MO, USA), and was used throughout this present work.

### 2.2. Sample Preparation

Clonal tea leaves were harvested from three varieties, including *Jinguanyin*, *Jinxuan*, and *Fudingdabai.* They were obtained from the Shenzhou Tea Comprehensive Experimental Base of the Tea Research Institute, Chinese Academy of Agricultural Sciences (Shengzhou City, Zhejiang Province). Fresh tea leaves were picked according to the standard that specifies one bud and one leaf. The manufacturing process for black tea was adapted from our previously used methods with a slight modification [14]. In brief, the harvested tea leaves were withered in bamboo sieves until the moisture reached around 62%, and they were then rolled using a machine (6CR-45, Shangyang Machinery Ltd., Hangzhou, China) at 38 rpm for 1.5 h. After rolling, tea leaves underwent fermentation in a machine (JY-6CHF-7, Jiayou Machinery Intelligent Technology Co., Ltd., Quanzhou, China) at 30 °C and a relative humidity of 90% for 3.5 h. A two-phase drying method was performed in this present work; the first drying was conducted at 120 °C until the moisture level reached 25%, followed by the second drying at 90 °C until the moisture content decreased to around 5% (JY-6CHZ-9B, Jiayou Machinery Intelligent Technology Co., Ltd., Quanzhou, China).

The method of blending fresh leaves has been used by many tea manufacturers, but scientific research based on chemical components is still lacking. Therefore, the treatments of this work were mainly based on practical experiences. Each variety of tea leaves was manufactured individually, resulting in three distinct black tea samples, which were labeled as JGY (*Jinguanyin* cooked), JX (*Jinxuan* cooked), and Fud (*Fudingdabai* cooked), respectively. For blending processing, according to our preliminary research, the ratio of fresh tea leaves used was 85% *Fudingdabai* to 15% *Jinguanyin* or *Jinxuan*. The blend of *Fudingdabai* with *Jinguanyin* cooked black tea was designated as FJG, while the blend with *Jinxuan* was labeled as FJX. Additionally, finished tea blends were created using the same ratio, combining 85% Fud with either 15% JGY or 15% JX, resulting in two blending samples named AFJG and AFJX, respectively. Therefore, a total of seven black tea samples were prepared based on the difference in varieties and blending methods (fresh leaf blending and finished tea blending). The sample names and prepared methods are summarized in Table 1. Among them, a single-variety treatment was used to compare the flavor differences between Fud, JGY, and JX. Blending fresh leaves treatment was used to confirm the contribution of adding oolong variety to flavor. As for the blending finished tea treatment, it was used to analyze the differences between blending fresh leaves and blending finished tea. All treatments are single-factor comparisons.

### 2.3. Sensory Evaluation

The sensory evaluation was conducted by 5 individuals (3 males and 2 females, aged between 20 and 60) who hold advanced or higher tea evaluation qualifications. Apart from being in good health, non-smokers, and not currently taking any medication, there were no other exclusion criteria. To eliminate the influence of environmental factors on the sensory evaluation results, the sensory testing was carried out in a single room with a temperature between 20–25 °C, and the evaluation was conducted in the morning (from 9:00 to 11:00). According to the Chinese national standard (GB/T23776-2018) “Methodology for Sensory Evaluation of Tea” [15], all the tea evaluators underwent four training sessions over a two-week period to select and identify sensory attributes for the sensory quality evaluation of black tea samples. Briefly, 3.00 ± 0.05 g of processed black tea was weighed, brewed with 150 mL of boiling water for 5 min, and prepared for evaluation by the group immediately. Each processed black tea sample was prepared in triplicate at room temperature and randomly coded, with each sensory participant tasting a total of 21 tea samples. Each sensory participant scored the appearance, liquor color, aroma, taste, and infused leaves of the black tea samples. A 100-point scale was used to evaluate the appearance (25%), infusion color (10%), aroma (25%), taste (30%), and infused leaf (10%) for each sample. All participants signed a written informed consent form before participating in the study, and there was no communication between the groups during the sensory evaluation process.

### 2.4. Quantification of Catechins, Gallic Acid, Caffeine, and Theaflavin

The contents of the caffeine, catechins, gallic acid (GA), and theaflavins were determined using high-performance liquid chromatography (HPLC, Agilent 1100VL, Agilent Technologies Inc., Santa Clara, CA, USA). Tea powder (0.2 g) was extracted using 5 mL of 70 °C methyl alcohol in a water bath maintained at 70 °C for 10 min, with shaking every 5 min. After cooling to room temperature, the mixture was centrifuged at 4000 rpm for 10 min. The residues were re-extracted using the same method, and supernatants were merged and diluted to 10 mL with methanol. The final extracted solutions were filtered through a 0.45 μm filter. A C18 column (5 μm, 4.6 mm × 250 mm; Agilent Technologies Inc., Shanghai, China) was used to separate the chemicals at a flow rate of 1.0 mL/min, and maintained at 40 °C. The injection volume was 10 μL, and the detector was set at 280 nm. The mobile phase consisted of A (100% acetonitrile) and B (2% acetic acid in water), with a gradient elution program as follows: mobile phase B was subjected to linear variation from 6.5 to 15% within 16 min, 15–25% at 16–25 min, 25–6.5% at 25–25.5 min, and maintained at 6.5% between 25.5 min and 30 min. For theaflavin analysis, the detector was adjusted at 380 nm, using the same gradient solutions. Mobile phase B was subjected to linear variation from 20–25% within 35 min, maintained at 25% at 35–38 min, and then from 25% to 20% at 38–40 min.

### 2.5. Amino Acids Analysis

Determination of amino acids was performed using a Sykam S-433D automatic amino acid analyzer equipped with an LCA K07/Li separation column (SYKAM, Munich, Germany). Ground samples (2.0 g) were extracted using 10 mL boiling water at 100 °C for 10 min, with continuous shaking. The supernatant was then centrifuged at 6000 rpm and 20 °C for 10 min, and subsequently filtered through a 0.45 μm aqueous membrane filter. The analysis conditions for the automatic amino acid analyzer referenced a previously reported method with a slight modification [16]. The gradient solvents included A (0.12 N, pH 2.9), B (0.3 N, pH 4.2), C (0.3 N, pH 8.0), and D (500 mM NaOH, 0.68 mM EDTA). The rate of elution and derivation flow were set at 0.45 mL/min and 0.25 mL/min, respectively. The column was maintained at 130 °C, and the injection volume was 50 μL. The gradient elution was as follows: 100% A (0–10 min); 79% A and 21% B (10–11 min); 79% A and 21% B (11–30 min); 62% A and 38% B (30–41 min); 100% B (41–63 min); 100% C (63–68 min); 100% C (68–78 min); 86% C and 14% D (78–81 min); 86% C and 14% D (81–83 min); 78% C and 22% D (83–83.1 min); 76% C and 24% D (83.1–95 min); 76% C and 24% D (95–101 min); 100% D (101–101.1 min); 100% D (101.1–106 min); 100% A (106–106.1 min); 100% A (106.1–129.8 min); 100% A (129.8–129.9 min). The mixture of amino acids standards was prepared to create external standard curves for qualitative and quantitative determination of amino acids in tea samples.

### 2.6. Volatiles Analysis Based on GC-MS

The ground tea sample (1.5 g) was placed into a 20 mL extraction vial containing 15 mL boiling water and 6 µL ethyl decanoate (100 mg/mL), and the vial was sealed with a cap immediately. The vials were moved onto an autosampler device (Agilent Technologies Inc., Bellefonte, PA, USA) and stirred in the thermostatic water base at 85 °C and 500 rpm for 20 min. The solid phase microextraction (SPME) fiber (DVB/CAR/PDMS, 50/30 µm, Supelco, Bellefonte, PA, USA) was inserted into the headspace to extract the volatile compounds for 30 min. Sample determination was performed in a GC-MS (Agilent 6890N-5973, Agilent Technologies Inc., Santa Clara, CA, USA) equipped with an Agilent HP-INNOWax capillary column (60 m × 250 μm × 0.25 μm). GC conditions were as follows: inlet temperature was 250 °C; gas interface temperature was 250 °C; The carrier gas was helium (>99.999%) with a constant flow rate of 1.5 mL/min and a split ratio of 4:1; heating program: initial 40 °C, hold for 5 min, 5 °C/min heating to 250 °C, hold for 10 min. MS conditions: ion source temperature was set at 230 °C, quadruple temperature was set at 150 °C, EI ionization energy was set at 70 eV, and full scan mode was performed with the scanning range of 35–550 *m*/*z*. The qualitative analysis was achieved using Agilent MassHunter Workstation B.08.01 Software Unknowns Analysis. Substance characterization was performed based on the retention index (RI), the NIS database, and internal standards.

### 2.7. Relative Odor Activity Value Analysis

The relative odor activity value (ROAV) was calculated using the method described by Yang et al. [17]. It could be calculated according to the equation ROAV = C/OT, where C is the relative concentration of a compound and OT is the corresponding odor threshold. Volatiles with ROAV > 1 were considered to have important contributions to the overall aroma.

### 2.8. Data Analysis and Statistics

All the samples were analyzed in triplicate, and the results were represented as mean values ± standard deviations. Partial least squares discriminant analysis (PLS-DA), S-plot analysis, and a permutation test for the taste and flavor components were conducted using Simca-p14.1 (Umetrics AB, Umea, Sweden). Statistical significance was evaluated using a one-way ANOVA test using SPSS 23.0 (IBM, Armonk, New York, NY, USA). A column diagram was performed in GraphPad Prism 9.5 (GraphPad Software, Inc., San Diego, CA, USA), a violin diagram and three-dimensional pie chart were generated in Origin 2021 (Origin Lab Corporation, Northampton, MA, USA), and a heat-map was achieved by circle heatmap (chiplot.online). All the software and websites we used are free and licensed.

## 3. Results and Discussion

### 3.1. Sensory Evaluation of Black Tea

**Figure 1:**
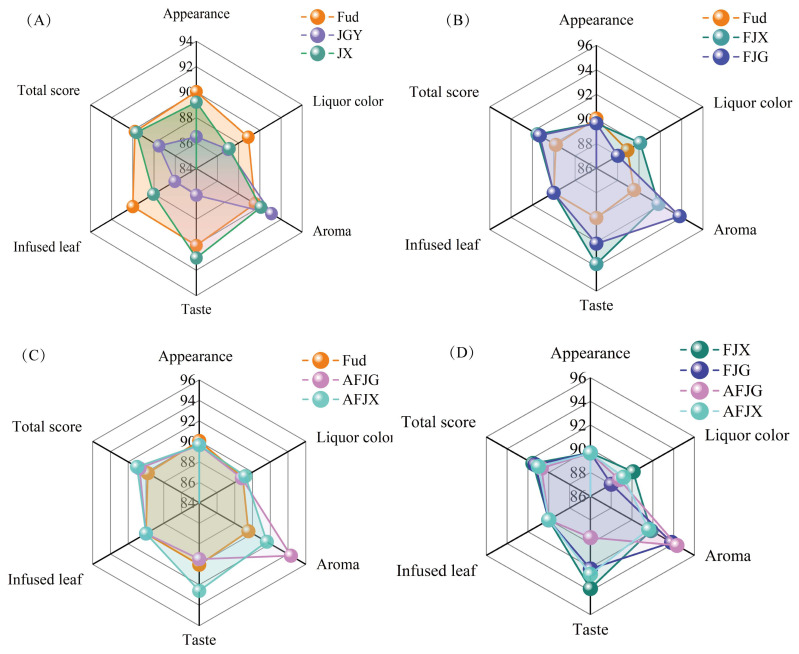
The scores of sensory evaluation results of three single varieties and different blending methods: (**A**) Comparison of sensory scores of black tea processed by Fud, JGY, and JX; (**B**) Comparison of sensory scores of black tea processed by Fud, FJG, and FJX; (**C**) Comparison of sensory scores of black tea processed by Fud, AFJG, and AFJX; (**D**) Comparison of sensory scores of black tea processed by FJG, FJX, AFJG, and AFJX.

The sensory evaluation results show that significant differences (*p* < 0.05) were observed among three black tea samples with a single variety. Fud presented better characteristics in terms of appearance, liquor color, and infused leaves, while JGY and JX scored much higher in aroma (Figure 1A). *Fudingdabai* is normally used as a control variety to select and compare the flavor differences between varieties, and its sensory quality is relatively balanced across the five sensory evaluation indices [18]. *Jinguanyin* and *Jinxuan* are oolong tea varieties normally used to make flowery-aroma black tea due to their outstanding floral characteristics [19,20]. When 15% of *Jinguanyin* or fresh *Jinxuan* tea leaves were added to *Fudingdabai*, resulting in the cooked black teas (FJG and FJX), aroma and taste were significantly enhanced, with minimal influence on the appearance and infused leaves compared with Fud (Figure 1B). The addition of oolong tea varieties in fresh leaves to the cooked tea products (FJG and FJX) presented a much stronger sweet and floral aroma as well as prolonged durability (Supplementary Materials Table S1). In addition, the richness of the taste was also enhanced via added oolong tea varieties in fresh leaves; FJG and FJX not only presented a mellow taste but were also endowed with sweet or fresh taste characteristics. The samples of finished tea blending (AFJG and AFJX) also had an improved sensory quality of the aroma compared with Fud (Figure 1C), though there was no difference (*p* < 0.05) in total scores between black tea samples made from fresh leaf blending and those made from finished tea blending (Figure 1D). Therefore, it could be considered that fresh leaf blending can enhance the sensory quality of black tea without any significant difference compared to the finished tea blending method.

### 3.2. Analysis of the Main Taste Compounds

The taste quality of black tea is influenced by its chemical composition, which includes catechins, caffeine, theaflavins, and amino acids [21]. To reveal the effects of blending methods on the taste quality of black tea, the contents and components of eight catechin monomers, gallic acid, caffeine, four theaflavin monomers, and twenty-one free amino acids were quantitatively compared in black tea samples. As shown in Supplementary Materials Figure S1A, Fud, JX, and JGY were grouped based on their taste compounds, indicating significant differences in the taste compounds among the samples. Similarly, in the Fud, FJX, and FJG samples (Supplementary Materials Figure S1C), blending fresh leaves during processing resulted in changes to the components and contents of the main taste compounds. Blended finished tea samples (AFJX and AFJG) also showed significant differences in their taste compounds. (Supplementary Materials Figure S1E). Moreover, black teas produced through fresh leaf blending exhibited different profiles compared to those from finished tea blending (Supplementary Materials Figure S1G). The effects of blending on the composition and contents of taste compounds in black tea samples will be further discussed in the following sections.

#### 3.2.1. Analysis of Catechins and Relevant Compounds

**Figure 2:**
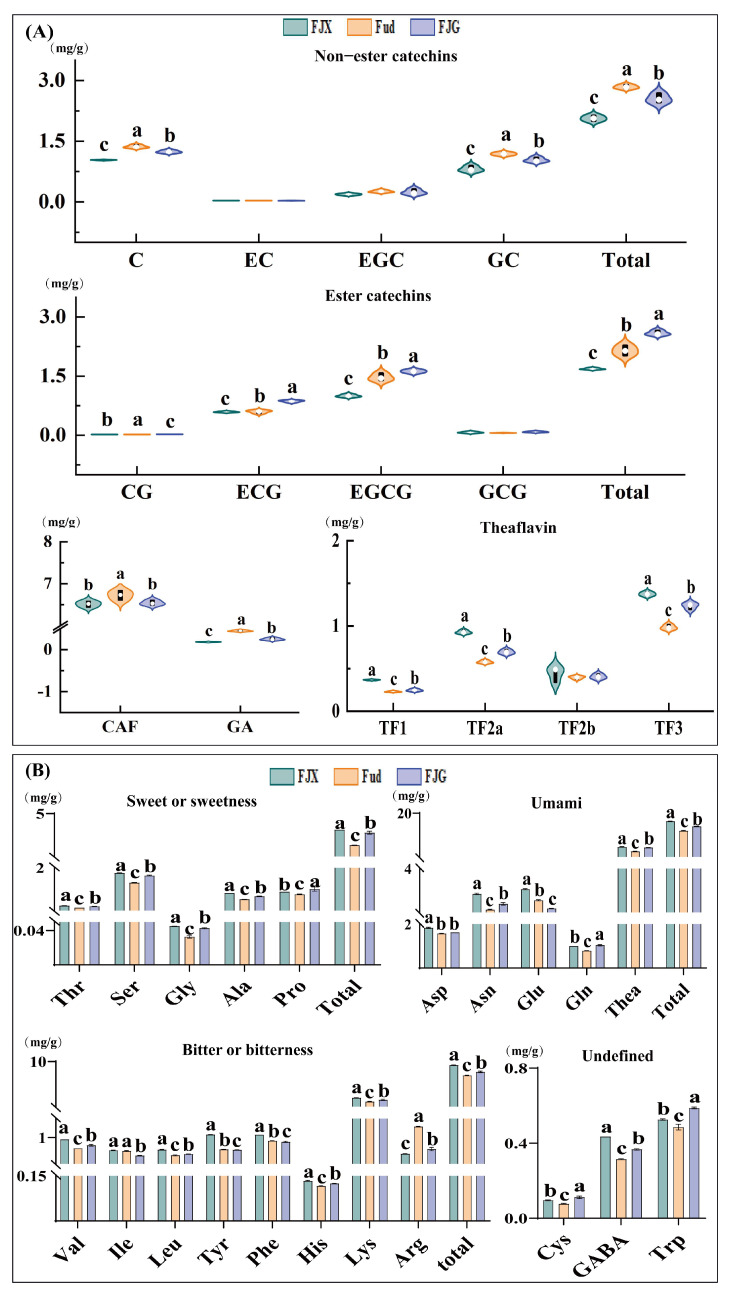
Comparative analysis of catechins, caffeine, gallic acid, theaflavins, and amino acids between fresh leaf blended black tea and Fud black tea: (**A**) Comparison of the contents of catechins, caffeine, gallic acid, and theaflavins in Fud, FJG, and FJX; (**B**) Comparison of the contents of amino acid components in Fud, FJG, and FJX. Note: Different letters indicate a statistically significant (*p* < 0.05) difference between groups.

Catechins are the main polyphenolic compounds found in tea and play important roles in determining the taste of black tea. Catechins can be classified based on their structure into two categories: non-ester catechins (C, EC, GC, EGC) and ester catechins (CG, ECG, GCG, EGCG) [22]. It was reported that ester catechins contribute to strong bitterness and astringency, while non-easter catechins provide a pleasant aftertaste in tea infusion [7]. In a comparative analysis, it was found that the content of ester catechins was highest in JGY, while the lowest levels of non-ester catechins were observed in JX (Supplementary Materials Figure S2A). When fresh tea leaves from *Fudingdabai* were blended with the *Jinxuan* variety, the contents of both ester-type catechins and non-ester catechins were significantly decreased (*p* < 0.05) in FJX (Figure 2A). However, the content of ester catechins increased in FJG while non-ester catechins decreased (Figure 2A). The increase in ester catechins in FJG and the decrease in FJX may be due to the higher content of ester-type catechins in JGY. Although the ester-type catechins in JX are higher than in Fud, after blending fresh leaves from different varieties, changes occur in the catechin content and ratio in the fresh leaves, as well as in the activities of PPO and POD, which promote the conversion of ester-type catechins into theaflavins in FJX [23]. The change in ester catechins between FJX and Fud was attributed to a significant decrease (*p* < 0.05) in the content of EGCG due to the addition of fresh *Jinxuan* leaves (Figure 2A). The high activity levels of polyphenol oxidase and peroxidase in fresh *Jinxuan* leaves led to the degradation of EGCG when blended with Fud [24]. In FJG samples, the increase in ester catechins corresponded with higher levels of ECG and EGCG, while the decrease in non-ester catechins was associated with declines in GC and C. Finished tea blending samples (AFJX and AFJG) presented relatively high levels of ester catechins (Supplementary Materials Figure S3A) compared to Fud, attributable to the addition of JX and JGY, which have high catechin contents. The ratio of ester catechins to non-ester catechins serves as an indicator of the strength of the tea infusion [25]. Therefore, blending treatments can adjust both the content and ratio of catechins, thereby affecting the strength of tea infusion. The difference is that blending fresh leaves involves complex biochemistry reactions, while blending finished tea is connected to the combination of the samples used.

Theaflavin is a key component used as a quality parameter in black tea [7]. It has an orange-red color and contributes briskness, strength, and brightness to black tea infusion [14]. The levels of theaflavin monomer contents in the JGY and JX samples were significantly higher than Fud (*p* < 0.05) (Supplementary Materials Figure S2A), indicating that oolong tea varieties have advantages in the formation of theaflavin during black tea processing. This may be due to the fact that different varieties determine the differences in the expression of polyphenol oxidase and peroxidase genes, leading to varying activation abilities of these enzymes during the tea processing [24], and that the blending of fresh leaves from two varieties causes changes in the expression of enzyme genes, promoting the conversion of catechins into theaflavins [26], which is consistent with the aforementioned research. This suggests that the addition of fresh leaves from oolong varieties can promote the accumulation and formation of theaflavins during black tea processing. Moreover, the content of theaflavins in AFJX and AFJG also showed a slight increase, although without statistical significance (Supplementary Materials Figure S3A). This further indicates that JGY and JX only provide a cumulative effect on the content of theaflavins in AFJX and AFJG. However, differences were observed between the content in fresh leaf blending samples and that in finished tea blending samples (Supplementary Materials Figure S4A). CAF, a natural purine alkaloid [27], is highly correlated with the bitterness intensity of tea soup [28,29]. GA contributes multiple taste characteristics, including astringency, sourness, and sweet aftertaste in tea soup [30]. The content of CAF and GA significantly decreased in FJX and FJG (*p* < 0.05) compared with Fud, confirming that blending with fresh leaves of oolong varieties can enhance the taste profiles during black tea processing, particularly by decreasing bitterness and astringency.

#### 3.2.2. Analysis of Amino Acids

Amino acids play a significant role in the umami, sweetness, and mellow taste of tea [31]. Amino acids can be categorized based on their contributions to taste profiles, which include umami, sweetness, and bitterness [32]. Research has indicated that aspartic acid, asparagine, glutamic acid, glutamine, and theanine are primarily responsible for the umami taste in tea infusion [33]. Therein, theanine is the most abundant amino acid in tea, constituting about 50–60% of the total amino acids present [29]. As shown in Supplementary Materials Figure S2B, the content of theanine was highest in the JX samples, while the theanine content in Fud was significantly higher (*p* < 0.05) than in JGY. The content of umami amino acids in FJX and FJG was significantly higher (*p* < 0.05) than that in Fud (Figure 2B), indicating that blending fresh leaves positively influences the umami taste profile. JX presented a higher content of theanine compared to Fud [34], suggesting mixing fresh *Fudingdabai* leaves with *Jinxuan* could enhance the levels of black tea after processing.

Sweet amino acids, including serine, glycine, alanine, and threonine, proline [33], were significantly increased in FJX and FJG compared with Fud (Figure 2B). This may be one of the reasons why FJG and FJX have a sweeter taste. Research shows that approximately 1.2% of the protein in tea leaves is broken down by peptidases, resulting in an increase in the amino acid content in the tea [35]. Meanwhile, blending fresh leaves of two different varieties during processing may cause changes in the expression of peptidase genes, promoting the breakdown of more proteins into amino acids. JX and JGY did not show consistent advantages in all sweet amino acids (Supplementary Materials Figure S2B). When blending finished tea, the addition of JX to Fud resulted in higher sweet amino acid content than the addition of JGY (Supplementary Materials Figure S3B), likely due to the higher amino acid content in JX (Supplementary Materials Figure S2B). Bitter amino acids include valine, leucine, isoleucine, tyrosine, phenylalanine, histidine, lysine, and arginine [33]. As shown in Supplementary Materials Figure S2B, JX exhibited the highest content of total bitter amino acids, while JGY had the lowest. The content of bitter amino acids was also elevated in FJX and FJG, consistent with the changes observed in sweet amino acids (Figure 2B). Although the levels of these bitter amino acids were relatively lower and their taste activity values were not high [36], they did not significantly influence the bitterness contribution to the teas. Moreover, valine, leucine, isoleucine, phenylalanine, lysine, and arginine can contribute to important aroma components via the Strecker degradation process in black tea [37]. GABA, cystine, and tryptophan are classified as undefined amino acids in terms of taste; these amino acids may not directly influence the taste of the infusion, but they can induce synergistic effects that enhance umami or inhibit bitterness and astringency. Blending fresh leaves during processing can adjust the contents and components of amino acids in black tea, meaning it could be a potential method to promote or regulate the taste flavor of black tea during processing.

### 3.3. Analysis of Volatile Components by GC-MS

Volatile compounds were determined using SPME-GC-MS to investigate the impact of different blending methods on aroma formation in black tea. A total of 97 volatile compounds were tentatively identified by comparing their mass spectra with those in the NIST and Wiley Libraries, and selecting compounds with a similarity index higher than 80. These volatiles were categorized into nine groups: 24 esters, 15 hydrocarbons, 19 alcohols, 10 aromatic hydrocarbons, nine heterocyclic compounds, eight aldehydes, six terpenes, four ketones, and two sulfides (Supplementary Materials Table S2, Figure S5). Esters, hydrocarbons, and alcohols were the three primary volatiles, accounting for 59.8% of all identified volatile compounds.

#### 3.3.1. Multiple Statistics Analysis in Volatiles

**Figure 3:**
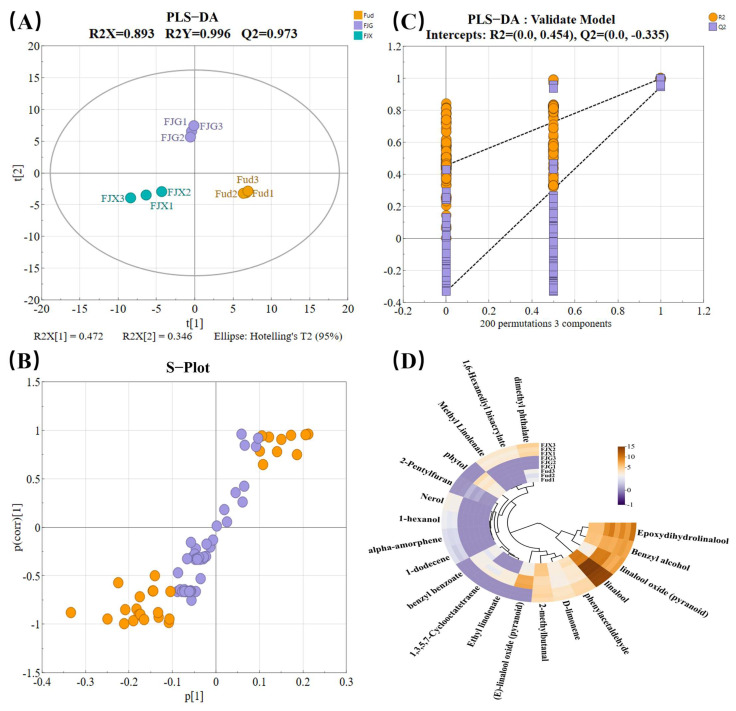
(**A**) Partial least squares discriminant analysis score diagram (PLS−DA) of volatile compounds in the blend of fresh leaves with black tea and Fud black tea; (**B**) Substitution test, orange—R2, purple—Q2; (**C**) Differential material screening s−plot; (**D**) Heat map of key different substance content distribution of the blend of fresh leaves with black tea and Fud black tea.

To explore the differences in volatile compounds of black tea samples concerning different varieties and blending methods, tea samples were analyzed by using the partial least squares discriminant analysis (PLS-DA). Before using PLS-DA, data standardization and missing value handling should be performed. As shown in Supplementary Materials Figure S6A, Fud, JX, and JGY were separated based on their volatile compounds. Similar results were also found in Fud, FJX, and FJG samples (Figure 3A), indicating that the addition of fresh leaves of oolong varieties (*Jinxuan* and *Jinguanyin*) to *Fudingdabai* resulted in noticeable changes in the volatile compounds of black tea after processing. The permutation test with 200 iterations was used to further evaluate the performance of the PLS-DA model in the present work. It was found that lower values for R2 and Q2 in the permutated models and the intercept of the Q2 regression line were less than 0 (Figure 3B). This strongly demonstrates that the PLS-DA model is not over-fitted and reliable. Moreover, AFJX and AFJG had significant differences in volatile compounds compared to Fud (Supplementary Materials Figure S7A), while AFJG and AFJX did not present large differences from FJX and FJG (Supplementary Materials Figure S8A). Therefore, it could be confirmed that the blending of fresh leaves during processing has a similar effect on the regulation of volatiles in black tea as blending finished teas.

Potential specific volatiles in the sample of fresh leaves blended during processing were identified by using S-plots, which displayed covariance (*p*) against correlation (*p*corr) (Figure 3C). The S-plot was used to identify key volatile compounds responsible for differences in the samples, and marked as orange circles. A total of 20 volatiles were screened as specific volatile candidates among Fud, FJX, and FJG (Table 2). Linalool oxide (pyranoid) was abundant in FJG, while D-limonene had abundant content in FJX, and Fud contained a higher level of 2-methylbutanal. To further identify key aroma-active volatiles based on their contribution to the overall aroma of the tea, a cutoff of ROAV > 1 was applied [17]. As a result, 12 volatiles were excluded for not meeting the cutoff values of ROAV, leaving eight volatiles as key aroma-active volatiles distinguishing Fud, FJX, and FJG (Figure 3D, Table 2). Similarly, 14 volatiles were identified as specific volatiles among the three single varieties of the cooked samples (Fud, JX, and JGY, Supplementary Materials Table S3), while six volatiles were noted for the finished tea blended samples (Fud, AFJX, and AFJG, Supplementary Materials Table S4).

#### 3.3.2. Key Aroma-Active Volatiles in Different Blending Treatments

According to our analysis of eight key aroma-active volatiles found in Fud, FJG, and FJX, we observed a significant increase (*p* < 0.05) in volatiles with floral characteristics in FJG or FJX samples (Figure 3D, Table 2). The volatile phenylacetaldehyde has a honey-like and sweet odor, contributing to the honey-like aroma profiles in tea samples [42]. In the present work, phenylacetaldehyde exhibited the highest ROAV and was significantly increased in FJX (Figure 3D, Table 2), which means phenylacetaldehyde played an important role in the formation of the honey-like aroma in FJX. Phenylacetaldehyde was found at a higher concentration in JX compared to Fud and JGY, indicating that it is a volatile characteristic of the Jinxuan variety. Additionally, the content of D-limonene was also significantly increased in FJX (*p* < 0.05), and presented the same change trend as phenylacetaldehyde (Figure 3D, Table 2). D-limonene has been reported as a key compound with a higher ROAV in the renowned Darjeeling black tea [43]. It can be considered that FJX contains a higher level of D-limonene, which is beneficial for improving aroma quality. 2-Pentylfuran exhibits a fruity aroma and is only present in FJG, indicating it might be an important contributor to the fruity aroma in FJG. The volatile 1-hexanol was only determined in FJX, linked to a sweet, fruity, and floral-like aroma. Investigation has shown that 1-hexanol significantly decreases during the fermentation process of black tea [44]; its presence in FJX may connect with complex enzymatic reactions. In addition, 1-hexanol is easily vaporized during the drying process in black tea and oolong tea due to its lower boiling point [45], making it difficult to retain in tea leaves after exposure to high temperatures during drying.

Benzyl alcohol and linalool oxide (pyranoid) are two floral-like aroma compounds in black tea, and their concentration was significantly increased (*p* < 0.05) in FJG and FJX. This may be an important reason for the prominent floral aroma in FJG and FJX. Benzyl alcohol can be formed through the hydrolysis of benzyl β-D-glucoside compounds [46], and is also derived from phenylpyruvic acid in tea leaves via enzyme reaction [47], meaning a high level of enzyme activity promotes the accumulation of benzyl alcohol. Investigation has identified benzyl alcohol as a key volatile in various teas with a sweet-floral profile [48,49]. Su et al. [50] confirmed that linalool and linalool oxide were dominant volatile components in Keemun black tea, with their content increasing alongside the grades of black tea [51]. It has been reported that linalool oxides originate from the glycoside forms of linalool in fresh tea leaves, rather than from the oxidization of linalool itself [37]. Linalool is not only sourced from glycosides but can also form from the degradation of carotenoids [52], with its content being influenced by the composition, distribution, and manufacture of carotenoids.

The volatile 2-methylbutanal is a volatile compound and a typical Strecker aldehyde derived from isoleucine, commonly found as an aroma compound in black teas. Three single-variety cooked black teas (Fud, JGY, and JX) contained a higher concentration of 2-methylbutanal, but its content was lower in FJG and FJX (Supplementary Materials Table S2). In contrast, linalool oxide (pyranoid), benzyl alcohol, and phenylacetaldehyde presented higher contents in JGY and JX, with an increasing trend in both FJG and FJX compared to Fud. The blending of fresh tea leaves during processing can enhance the content of floral-like aroma compounds when blended with tea of the oolong variety. Except for linalool and linalool oxide (pyranoid), blending finished tea (AFJG and AFJX) led to an increase in the content of methyl salicylate and geraniol compared with Fud (Supplementary Materials Table S4), both of which were also present at a higher level at JGY and JX (Supplementary Materials Table S3). Therefore, both blending fresh leaves and blending finished tea can improve the aroma quality of black tea by increasing the content of sweet and floral aroma compounds. However, blending fresh leaves has a greater impact on enhancing the flavor of black tea, while blending finished tea has a more noticeable effect on the aroma. The differences in aroma compounds result in their aroma characteristics not being entirely consistent.

## 4. Conclusions

The present work examined the effect of the blending of fresh leaves during processing on the flavor quality of black tea. The results indicated that blending fresh leaves during processing can improve and adjust the aroma and taste quality of black tea. Compared to blended finished tea, blending fresh leaves results in a greater enhancement of the taste (fresh and mellow) of black tea. However, there were no significant differences in the overall scores for appearance, aroma, taste, liquor color, and infused leaves. The results show that blending fresh leaves during processing not only avoids the time-consuming and labor-intensive blending steps and high costs, but also improves and adjusts the aroma and taste quality of the black tea. In addition, blending *Fudingdabai* with fresh *Jinguanyin* or *Jinxuan* leaves resulted in black tea with higher concentrations of floral aroma compounds, including phenylacetaldehyde, linalool oxide (pyranoid), benzyl alcohol, and linalool. Therefore, blending fresh leaves during processing could be a potential method to enhance and improve the quality of black tea, but there is still a lack of research on the mechanisms of blending fresh leaves. This study lays a solid foundation for the industrial efficient production of high-quality black tea.

## Figures and Tables

**Table 1:** Samples’ preparation and their manufacture methods.

No.	Name	Treatments	Manufacture Methods
1	Fud	Single variety, without blending	Using the variety of *Fudingdabai* manufactured black tea.
2	JGY	Single variety, without blending	Using the variety of *Jinguanyin (oolong tea variety)* manufactured black tea.
3	JX	Single variety, without blending	Using the variety of *Jinxuan (oolong tea variety)* manufactured black tea.
4	FJG	Mixed varieties, fresh leaf blending	Fresh leaves were blended with a ratio of 85% *Fudingdabai* and 15% *Jinguanyin*.
5	FJX	Mixed varieties, fresh leaf blending	Fresh leaves were blended with a ratio of 85% *Fudingdabai* and 15% *Jinxuan*.
6	AFJG	Mixed varieties, finished tea blending	Fresh leaves were not blended; samples manufactured by blending with the ratio of 85% Fud and 15% JGY.
7	AFJX	Mixed varieties, finished tea blending	Fresh leaves were not blended; samples manufactured by blending with the ratio of 85% Fud and 15% JX.

**Table 2:** Odor descriptions and ROAV of key differentiating volatiles after mixing fresh leaves with black tea and fud black tea.

Compounds	OT (mg/kg)	Odor Descriptor	ROAV			*p*
			Fud	FJG	FJX	
2-methylbutanal		cocoa, almond (*)	43.28	31.76	34.24	<0.05
D-limonene	0.004	mint, lemon, citrus, orange, fresh, sweet (*, #)	46.25	37.75	80.25	<0.05
linalool	0.028	lemon, citrus, orange, floral, sweet, woody, blueberry, lavender, flower, green (*, #)	35.99	54.07	50.05	<0.05
phenylacetaldehyde	0.002	hyacinth, honey, clover, sweet, cocoa, grapefruit, green, peanut, floral, bitter (*, #)	162.50	131.50	213.00	<0.05
linalool oxide (pyranoid)	0.19	flower (*)	0.93	5.19	3.95	<0.05
benzyl alcohol	0.62	berry, balsamic, rose, floral, walnut, sweet, cherry, phenolic, flower, grapefruit (*, #)	0.92	1.15	1.25	<0.05
1-hexanol	0.0056	oil, ethereal, resin, fusel, sweet, fruity, flower, green (*, #)	0.00	0.00	15.00	<0.05
2-Pentylfuran	0.0058	fruity (#)	0.57	4.14	0.86	<0.05
Epoxydihydrolinalool	n. f.	flower, wood (*)	-	-	-	<0.05
dimethyl phthalate	n. f.	n. f.	-	-	-	<0.05
1,3,5,7-cyclooctatetraene	n. f.	n. f.	-	-	-	<0.05
ethyl linolenate	n. f.	fruit (*)	-	-	-	<0.05
α-amorphene	n. f.	n. f.	-	-	-	<0.05
1-dodecene	n. f.	n. f.	-	-	-	<0.05
1,6-Hexanediyl bisacrylate	n. f.	n. f.	-	-	-	<0.05
methyl Linolenate	450	oily, fatty, woody (*)	0.00	0.00	0.00	<0.05
(E)-linalool oxide (pyranoid)	3	woody, tea-like (#)	0.05	0.25	0.00	<0.05
phytol	0.64	powdery, delicate, waxy, balsam, flower (*, #)	0.28	0.49	0.00	<0.05
benzyl benzoate	0.341	oil, pineapple, balsamic, herbal, strawberry, oily, faint, sweet, balsam, cherry, almond, herb, cheese (*, #)	0.28	0.37	0.00	<0.05
nerol	0.29	sweet (*)	0.00	0.00	0.43	<0.05

Note: OT, odor thresholds; all odor thresholds were obtained from “Compilations of odor thresholds values in air, water and other media” written by [38], or obtained from the relevant literature of [39,40,41]. Odor descriptions are found on the following websites: *: https://www.flavornet.org/flavornet.html (accessed on 1 November 2023); #: https://cosylab.iiitd.edu.in/flavordb/search (accessed on 1 November 2023); “n. f.”: Data were not found in the literature.

## Data Availability

The original contributions presented in the study are included in the article/Supplementary Material, further inquiries can be directed to the corresponding authors.

## Supplementary Materials

The following supporting information can be downloaded at: https://www.mdpi.com/article/10.3390/foods14060941/s1, Table S1: Sensory evaluation of congou black tea under different blending treatments. Table S2: Characterization of volatile compounds in mix black tea based on GC-MS. Table S3: Odor Threshold, Description, and ROAV of Single-Component Volatile Compounds. Odor descriptions and ROAV of key differentiating volatiles of three single variety. Table S4: Odor descriptions and ROAV of key differentiating volatiles of finished tea blended with black tea. Table S5: Odor descriptions and ROAV of key differentiating volatiles of finished tea blended with black tea and fresh leaves blended with black tea. Figure S1: Partial least squares discriminant analysis score chart of main tast components in blending black tea and its raw materials and substitution test. Figure S2: Analysis of the main chemical components (catechins, caffeine, gallic acid, theaflavins and amino acids between of three single varieties of black tea. Note: different letters indicated statistically significant (*p* < 0.05) between groups. Figure S3: Comparative analysis of the main chemical components (catechins, caffeine, gallic acid, theaflavins and amino acids) of finished tea and Fud black tea. Note: different letters indicated statistically significant (*p* < 0.05) between groups. Figure S4: Comparative analysis of the main chemical components (catechins, caffeine, gallic acid, theaflavins and amino acids) of finished tea blended with black tea and fresh leaves blended with black tea. Note: different letters indicated statistically significant (*p* < 0.05) between groups. Figure S5: Classification of volatile organic compounds in different blending black tea and its raw materials. Figure S6: (**A**) Partial least squares discriminant analysis score diagram (PLS-DA) of volatile compounds in three single varieties of black tea. (**B**) Substitution test. (**C**) Differential material screening s-plot. (**D**) Heat map of different key substance content distribution of three single varieties. Figure S7: (A) Partial least squares discriminant analysis score diagram (PLS-DA) of volatile compounds in finished black tea blended and Fud black tea. (**B**) Substitution test. (**C**) Differential material screening s-plot (**D**) Heat map of different key substance content distribution of finished black tea blended and Fud black tea. Figure S8: (A) Partial least squares discriminant analysis score diagram (PLS-DA) of volatile compounds in finished tea blended with black tea and fresh leaves blended with black tea. (**B**) Substitution test. (**C**) Differential material screening s-plot. (**D**) Heat map of different key substance content distribution of finished tea blended with black tea and fresh leaves blended with black tea.
