# Supplementary material for: Improving the Sensory Quality of Black Tea by Blending Varieties During Processing
Source: Foods. 2025 Mar 10;14(6):941. doi: 10.3390/foods14060941 (PMC11941214; doi:10.3390/foods14060941)
Supplement: Supplementary file 1 [file foods-14-00941-s001.zip › foods-3465935-supplementary.pdf]

SUPPLEMENTARY MATERIALS FOR

Improving the sensory quality of black tea by blending varieties during processing

Wenxue Chen[a,b], Jiezhong Zan[b], Linfen Yan[c], Haibo Yuan[b], Peiqiang Wang[a*], Yongwen Jiang[b*], Hongkai Zhu[b*]

[a] College of Horticulture, Qingdao Agricultural University, Qingdao, Shandong, 266109, China

[b] Tea Research Institute, China Academy of Agricultural Sciences, Key Laboratory of Tea Biology and Resource Utilization, Ministry of Agriculture, Hangzhou 310008, China.

[C] Sichuan Tea Industry Group Co., Ltd, Yibin 644000, China.

*Corresponding author:

Peiqiang Wang, tel.:15725222118, E-mail: wpqtea@163.com

Yongwen Jiang, tel.:13957114526, E-mail: jiangyw@tricaas.com

Hongkai Zhu, tel.: +86 0571 85102505, E-mail: hongkai.zhu@hotmail.com

Table S1. Sensory evaluation of congou black tea under different blending treatments.

Name of sample	Appearance（25%）		Liquor color（10%）		Aroma（25%）		Taste（30%）		Infused leaf（10%）		Total score
	Comment	Score	Comment	Score	Comment	Score	Comment	Score	Comment	Score	
Fud	tight and heavy, curly, with tippy, black auburn	90.04±0.25a	fairly red，slightly bright	88.91±0.29b	sweet with slightly flowery, strong and lasting	89.54±0.23f	sweet and mellow, slightly fresh	90.05±0.31d	tender, thick, even red and bright	90.00±0.5a	89.8±0.11b
JGY	coarse and bold, bent, with flat and lumpy, black auburn	86.5±0.27c	red, nearly bright	87±0.21d	flowery and fruity, strong and lasting	91.10±0.18d	heavy and strong	86.10±0.45e	slightly tend, thick, dark red, slightly green	86.03±0.12b	87.53±0.16c
JX	tight and heavy, curly, with fair tippy, black auburn	89.21±0.17b	orange-red, nearly bright	87.12±0.35d	highly sweet with flowery aroma, strong and lasting	90.10±0.47e	sweet and slightly mellow, fairly fresh	91.03±0.26c	slightly tend, thick, slightly red and bright	88.05±0.2b	89.65±0.28b
FJX	tight and heavy, curly, with tippy, black auburn	89.66±0.16a	fairly red, bright	90.11±0.19a	higher sweetness with flowery aroma, strong and lasting	91.83±0.21c	sweet and mellow, fairly fresh	93.80±0.19a	tender, thick, even red and bright	90.00±0.07a	91.52±0.14a
FJG	tight and heavy, curly, with tippy, black auburn	89.65±0.22a	red, slightly bright	88.01±0.25c	highly sweet and flowery with slightly fruity, strong and lasting	93.81±0.28b	mellow and thick, fairly fresh	92.14±0.41b	tender, thick, even red and bright	90.01±0.09a	91.31±0.2a
AFJG	tight and heavy, curly, with tippy, black auburn	89.63±0.34a	fairly red，slightly bright	88.8±0.29b	highly sweet and flowery with fruity, strong and lasting	94.32±0.29a	mellow and thick	89.5±0.27d	tender, thick, even red and bright	90±0.1a	90.72±0.3a
AFJX	tight and heavy, curly, with tippy, black auburn	89.64±0.18a	fairly red，slightly bright	89.2±0.23b	higher sweetness with flowery aroma, strong and lasting	91.61±0.28c	sweet and mellow, fresh	92.6±0.32b	tender, thick, even red and bright	90.00±0.2a	91.01±0.2a

Note: Different small letters in the same column mean the samples are significantly different at $P < 0.05$.

Table S2. Characterization of volatile compounds in mix black tea based on GC-MS.

Compounds	CAS	RT	RI[a]	RI[b]	Contents (mg/kg)						
					Fud	JGY	JX	FJG	FJX	AFJX	AFJG
dimethyl sulfide	75-18-3	3.97	757	773	7.72±0.23	7.32±0.61	8.03±1.65	8.68±1.62	7.17±2.26	7.33±0.33	7.45±0.19
isobutyraldehyde	78-84-2	4.47	819	842	6.5±0.16	3.06±0.28	6.43±1.01	3.52±1.03	3.98±0.98	5.38±0.38	6.09±0.36
2-methylbutanal	96-17-3	6.05	911	936	5.41±0.05	6.55±1.26	0.00	3.97±0.17	4.28±0.32	5.39±0.09	6.54±0.74
D-limonene	138-86-3	13.97	1203	1224	1.85±0.07	3.56±1.21	5.48±0.79	1.51±0.04	3.21±0.21	2.44±0.19	1.81±0.23
trans-2-hexenal	6728-26-3	15.11	1232	1250	1.28±0.02	1.37±0.45	1.29±0.57	1.32±0.06	1.27±0.22	1.14±0.14	0.87±0.11
3,5-dimethyldodecane	107770-99-0	19.47	1285	1268	0.68±0.5	0.00	0.00	0.00	0.00	0.00	0.00
(Z)-hex-3-en-1-ol	928-96-1	19.80	1371	1398	1.55±0.07	5.48±2.1	1.86±0.62	1.78±0.42	2.16±0.59	2.15±0.84	2.69±0.38
2,4,6-Collidine	108-75-8	19.94	1360	1384	1.03±0.46	0.00	0.00	1±0.1	1±0.3	1.03±0.47	1±0.12
nonanal	124-19-6	20.14	1403	1419	0.84±0.23	0.00	0.00	0.00	0.00	0.00	0.00
Epoxydihydrolinalool	1365-19-1	22.26	1473	1490	5.97±0.03	27.17±5.9	6.47±2.21	8.87±1.11	9.3±1.3	8.65±0.65	14.13±0.43
3-furaldehyde	498-60-2	22.14	1483	1499	0.19±0.02	0.00	0.00	0.00	0.00	0.51±0.21	0.00
benzaldehyde	100-52-7	23.76	1502	1526	3±0.56	2.79±0.99	3.57±0.58	2.62±0.71	2.85±0.85	2.74±0.74	3.68±0.5
linalool	78-70-6	24.08	1552	1568	10.08±0.13	15.69±0.59	14.8±0.13	15.14±0.18	14.01±0.21	12.37±0.28	12.81±0.19
dimethyl sulfoxide	67-68-5	25.54	1579	1595	1.64±0.28	0.00	1.62±0.46	0.00	0.00	2.32±0.32	0.00
beta-cyclocitral	432-25-7	26.08	1638	1654	0.69±0.01	0.00	0.98±0.07	0.47±0.07	0.00	0.00	0.86±0.13
phenylacetaldehyde	122-78-1	26.66	1663	1650	3.25±0.57	2.17±0.17	3.77±1.06	2.63±0.21	4.26±0.12	2.58±0.58	3.04±0.12
trans-2-hexenyl hexanoate	53398-86-0	27.10	1668	1664	0.82±0.07	0.00	0.00	0.71±0.09	0.96±0.15	0.00	0.00
(E)-linalool oxide (pyranoid)	39028-58-5	28.69	1770	1783	1.39±0.31	5.4±0.71	0.00	7.37±0.17	0.00	0.00	0.00
linalool oxide (pyranoid)	14049-11-7	29.18	1721	1700	1.77±0.39	12.14±2.31	4.77±0.24	9.87±0.11	7.51±0.51	4.81±0.27	3.12±0.08
methyl salicylate	119-36-8	29.76	1756	1778	4.1±0.05	21.53±3.67	4.56±0.99	6.25±0.25	6.43±0.43	5.94±0.94	9.82±0.39
trans-Anethole	4180-23-8	30.81	1847	1859	1.33±0.47	1.96±0.24	1.14±0.33	0.00	0.00	1.26±0.1	1.43±0.16
Geraniol	106-24-1	30.99	1857	1850	7.09±0.75	39.4±5.43	27.29±4.87	9.33±1.1	9.49±1.49	11.56±1.56	16.13±0.67
Benzyl alcohol	100-51-6	31.68	1898	1996	5.72±0.09	15.74±3.71	5.72±0.88	7.16±0.14	7.73±0.31	5.71±0.2	10.26±0.26
2-phenylethanol	60-12-8	32.45	1935	1950	29.33±0.32	22.37±3.59	8.21±2.41	29.08±2.3	34.86±4.9	30.19±4.86	28.69±0.28
2-phenyl-2-butenal	4411-89-6	33.01	1933	1949	0.32±0.1	0.00	0.00	0.33±0.09	0.49±0.09	0.34±0.14	0.55±0.14
beta-Jonone	14901-07-6	33.17	1964	1976	0.78±0.08	0.00	1.18±0.44	0.93±0.14	1.02±0.1	0.94±0.12	1.39±0.09
methyl myristate	124-10-7	34.43	2014	2016	0.34±0.03	0.46±0.14	0.00	0.37±0.14	0.34±0.09	0.34±0.07	0.93±0.22
Methyl hexadecanoate	112-39-0	38.39	2208	2224	6.39±0.38	11.28±2.93	11.28±2.22	8.92±0.98	8.03±1.78	8.46±0.23	14.04±0.43
2,4-Di-tert-butylphenol	96-76-4	39.86	2315	2336	0.63±0.14	1.35±0.12	0.00	0.00	0.00	0.00	1.06±0.51
dimethyl phthalate	131-11-3	39.96	2325	2347	0.9±0.21	0.00	0.00	0.00	4.4±0.5	1.09±0.09	0.00
galaxolide	1222-05-5	40.38	1913	1930	0.27±0.15	0.00	0.00	0.00	0.00	0.00	0.00
Methyl stearate	112-61-8	42.04	2434	2451	0.81±0.3	1.24±0.13	0.88±0.09	0.00	0.75±0.05	0.89±0.09	0.00
elaidic acid methyl ester	1937-62-8	42.41	2445	2461	5.92±0.41	7.66±1.37	5.27±0.71	5.1±0.47	4.71±0.71	4.68±1.59	9.16±0.29
6-hydroxynaphthalene-1,2-dione	607-20-5	43.04	1712	1747	0.55±0.17	0.00	0.00	0.00	0.00	0.00	0.00
methyl linoleate	112-63-0	43.21	2509	2511	2±0.45	2.66±0.87	2.72±0.7	2.24±0.28	2.3±0.6	2.61±0.61	4.81±0.4
Methyl cis-11,14,17-eicosatrienoate	55682-88-7	44.30	2241	2245	2.35±0.25	3.85±1.26	0.00	0.00	0.00	0.00	6.46±1.02
phytol	150-86-7	45.05	2622	2631	1.8±0.18	8.45±1.81	0.00	3.14±0.76	0.00	1.69±0.06	3.56±0.12
benzyl benzoate	120-51-4	45.53	2655	2653	0.94±0.18	0.00	0.00	1.26±0.26	0.00	0.93±0.17	0.92±0.13
4-methyldodecane	6117-97-1	13.34	1258	1276	0.00	0.00	0.00	0.00	0.00	0.00	1.15±0.65
2-hexenal	505-57-7	15.13	1213	1239	0.00	0.00	0.00	0.00	0.00	0.00	0.87±0.13
2-Pentylfuran	3777-69-3	15.21	1234	1219	0.03±0.01	0.42±0.04	0.05±0.01	0.24±0.08	0.05±0.01	0.04±0.01	0.2±0.03
styrene	100-42-5	16.15	1262	1255	2.46±0.31	2.25±0.2	2.51±0.21	2.59±0.25	2.49±0.15	2.21±0.11	2.45±0.43
cis-2-penten-1-ol	1576-95-0	17.99	1334	1341	0.00	0.00	2.75±0.51	1.58±0.58	1.75±0.85	0.00	2.2±0.37
Furfural	98-01-1	22.13	1482	1465	0.00	0.43±0.15	0.00	0.42±0.01	0.00	0.00	0.68±0.08
(Z)-3-hexen-1-yl caproate	31501-11-8	26.79	1662	1675	0.00	3.02±0.21	0.00	1.84±0.35	1.95±0.39	2.13±0.54	2.1±0.32

Anethole	104-46-1	30.81	1815	1839	0.00	0.00	0.00	1.62±0.62	1.68±0.68	0.00	2.42±0.55
3-phenylfuran	13679-41-9	0.00	1880	1869	0.00	0.00	0.00	0.00	0.00	0.00	1.08±0.2
cis-Jasmone	488-10-8	0.00	1969	1963	0.00	0.00	0.00	0.00	0.00	0.00	0.38±0.09
Nerolidol	7212-44-4	34.95	2044	2058	0.00	2.75±0.7	0.00	1.35±0.35	1.49±0.49	0.00	0.31±0.04
ethyl palmitoleate	56219-10-4	0.00	2266	2260	0.00	0.00	0.00	0.00	0.00	0.00	1.15±0.53
Methyl Pentadecanoate	7132-64-1	0.00	2099	2089	0.00	0.00	0.00	0.00	0.00	0.00	2.04±0.82
2-methoxyoxolane	13436-45-8	8.62	726	739	0.00	0.00	1.05±0.07	0.6±0.14	0.00	0.00	0.00
1,3,5,7-Cyclooctatetraene	629-20-9	16.12	1199	1210	0.00	0.00	1.99±0.77	1.45±0.7	0.00	0.00	0.00
3-methyltridecane	6418-41-3	19.51	1366	1369	0.00	0.00	0.00	1.09±0.09	0.9±0.12	0.00	0.00
(Z)-tetradec-3-ene	41446-67-7	24.33	1384	1389	0.00	0.00	0.00	0.4±0.11	0.00	0.00	0.00
3-Methylpentadecane	2882-96-4	24.76	1574	1560	0.00	0.00	0.00	0.56±0.06	0.65±0.05	0.00	0.00
alpha-amorphene	483-75-0	28.42	1679	1692	0.00	0.00	0.00	0.8±0.18	0.00	0.00	0.00
alpha-curcumene	644-30-4	29.46	1786	1794	0.00	1±0.11	0.31±0.05	1.22±0.22	1.05±0.13	0.47±0.13	0.00
cuparene	16982-00-6	30.64	1849	1862	0.00	0.36±0.13	0.00	0.67±0.07	0.6±0.08	0.00	0.00
Methylionene	31197-54-3	32.59	1925	1945	0.00	0.00	0.00	1.07±0.16	0.00	0.00	0.00
3,5-Di-tert-butylphenol	1138-52-9	39.86	2328	2311	0.00	0.00	0.00	0.93±0.03	0.00	0.00	0.00
Methyl 16-methylheptadecanoate	5129-61-3	42.04	2103	2131	0.00	0.00	0.00	0.86±0.04	0.00	0.00	0.00
Ethyl linolenate	1191-41-9	44.30	2613	2620	0.00	0.00	4.39±1.15	2.08±0.08	0.00	2.71±0.58	0.00
gamma-terpinene	99-85-4	15.40	1255	1235	0.00	0.00	0.67±0.06	0.00	0.54±0.13	0.54±0.15	0.00
p-cymene	99-87-6	16.26	1261	1275	0.00	0.00	0.00	0.00	0.4±0.06	0.00	0.00
1-hexanol	111-27-3	18.94	1361	1381	0.00	0.00	0.00	0.00	0.84±0.05	0.00	0.00
Cyclooctane	292-64-8	24.33	1014	1032	0.00	0.00	0.00	0.00	0.38±0.07	0.00	0.00
α-amorphene	20085-19-2	28.42	1670	1685	0.00	0.00	0.00	0.00	0.64±0.04	0.00	0.00
Nerol	106-25-2	29.99	1808	1826	0.00	0.00	0.00	0.00	1.24±0.24	0.00	0.00
N-ethylsuccinimide	2314-78-5	31.96	1836	1841	0.00	0.00	0.00	0.00	0.55±0.15	0.00	0.00
3,7-dimethyl-octa-1,5-diene-3,7-diol	13741-21-4	33.09	1961	1963	0.00	0.00	0.00	0.00	0.38±0.12	0.00	0.00
1-dodecene	112-41-4	33.49	1241	1244	0.00	0.00	0.00	0.00	0.6±0.12	0.00	0.00
1-Phenoxy-2-propanol	770-35-4	35.13	1246.4	1264	0.00	0.00	0.00	0.00	0.42±0.13	0.00	0.00
1,6-Hexanediyl bisacrylate	13048-33-4	38.47	1604	1619	0.00	0.00	0.00	0.00	2.43±0.43	0.00	0.00
Methyl Linolenate	301-00-8	44.30	2583	2599	0.00	0.00	0.00	0.00	2.41±0.41	0.00	0.00
methyl hex-3-enoate	13894-62-7	16.04	1252	1268	0.00	0.00	0.00	0.00	0.00	1.31±0.22	0.00
Propylcyclopropane	2415-72-7	18.95	609	634	0.00	0.00	0.00	0.00	0.00	0.86±0.11	0.00
(*E*)-2-Hexen-1-ol	928-95-0	20.39	1412	1425	0.00	2.76±0.35	0.00	0.00	0.00	2.36±0.14	0.00
1-Octanol	111-87-5	24.31	1550	1570	0.00	0.00	0.00	0.00	0.00	0.35±0.04	0.00
cis-10-Heptadecenoic Acid methyl ester	75190-82-8	38.89	1986	1998	0.00	0.00	0.00	0.00	0.00	0.51±0.14	0.00
(4-tert-butylphenyl)trimethylsilane	18412-68-5	39.85	1229	1253	0.00	0.00	0.00	0.00	0.00	0.61±0.1	0.00
Staflex BOP	84-78-6	46.49	2317	2332	0.00	0.00	0.00	0.00	0.00	2.53±0.36	0.00
1,3-dimethyl-2-ethylbenzene	2870-04-4	16.34	1372	1356	0.00	0.58±0.16	0.00	0.00	0.00	0.00	0.00
(-)-β-caryophyllene	87-44-5	25.47	1623	1620	0.00	0.66±0.23	0.00	0.00	0.00	0.00	0.00
dehydrolinalool	29957-43-5	25.64	1621	1644	0.00	1.51±0.39	0.00	0.00	0.00	0.00	0.00
farnesene	502-61-4	28.86	1758	1742	0.00	1.26±0.17	0.00	0.00	0.00	0.00	0.00
beta-calacorene	50277-34-4	32.61	1918	1936	0.00	1.12±0.27	0.00	0.00	0.00	0.00	0.00
3-Phenoxy-1-propanol	6180-61-6	35.13	2063	2032	0.00	0.42±0.11	0.00	0.00	0.00	0.00	0.00
4-Phenyltoluene	644-08-6	36.56	2117	2130	0.00	0.96±0.29	0.00	0.00	0.00	0.00	0.00

alpha-cadinol	481-34-5	37.89	2235	2221	0.00	0.72±0.14	0.00	0.00	0.00	0.00	0.00
4-ethyl-m-xylene	874-41-9	16.25	1348	1362	0.00	0.00	0.49±0.08	0.00	0.00	0.00	0.00
Methyl laurate	111-82-0	30.11	1815	1830	0.00	0.00	0.35±0.09	0.00	0.00	0.00	0.00
octyl octanoate	2306-88-9	34.50	2020	2026	0.00	0.00	1.08±0.34	0.00	0.00	0.00	0.00
(-)-α-CUBEBENE	17699-14-8	38.09	1468	1479	0.00	0.00	5.24±0.13	0.00	0.00	0.00	0.00
gamma-eudesmol	1209-71-8	38.60	2185	2120	0.00	0.00	1.29±0.25	0.00	0.00	0.00	0.00
beta-Eudesmol	473-15-4	38.75	2257	2271	0.00	0.00	1.45±0.3	0.00	0.00	0.00	0.00
Methyl (9Z)-9-hexadecenoate	1120-25-8	38.89	2245	2239	0.00	0.00	0.77±0.06	0.00	0.00	0.00	0.00

Note: data were expressed as mean ± SD (n = 3). RT represented retention time. RI^a represented retention indices referred to the literature value with HP-INNOWAX column or equivalent chromatographic column [NIST Chemistry WebBook (http://webbook.nist.gov/chemistry/), https://www.chemspider.com and http://www.flavornet.org/flavornet.html/]. RI^b represented the linear retention indices calculated from a series of n-alkanes (C7-C40).

Table S3. Odor Threshold, Description, and ROAV of Single-Component Volatile Compounds. **Odor descriptions and ROAV of key differentiating volatiles of three single variety.**

Compounds	OT (mg/kg)	Odor Descriptor	ROAV			P
			Fud	JGY	JX	
isobutyraldehyde	0.0435	green, malt, fresh, floral, pungent, aldehydic (*, #)	14.94	7.03	14.78	< 0.05
2-methylbutanal	0.0125	cocoa, almond (*)	43.28	52.40	0.00	< 0.05
(Z)-hex-3-en-1-ol	0.0039	leafy, grass, foliage, vegetable, herbal, fresh, cut grass, oily, green (*, #)	39.74	140.51	47.69	< 0.05
phenylacetaldehyde	0.002	hyacinth, honey, clover, sweet, cocoa, grapefruit, green, peanut, floral, bitter (*, #)	162.50	108.33	188.50	< 0.05
linalool oxide (pyranoid)	0.19	flower (*)	0.93	6.39	2.51	< 0.05
methyl salicylate	0.04	mint, wintergreen, peppermint (*, #)	10.25	53.83	11.40	< 0.05
Geraniol	0.075	citrus, rose, waxy, floral, sweet, fruity, geranium (*, #)	9.45	52.53	36.39	< 0.05
Benzyl alcohol	0.62	berry, balsamic, rose, floral, walnut, sweet, cherry, phenolic, flower, grapefruit (*, #)	0.92	2.54	0.92	< 0.05
2-phenylethanol	0.14	lilac, honey, rose flower, floral, spice, bitter, rose dried (*, #)	20.95	15.98	5.86	< 0.05
phytol	0.64	powdery, delicate, waxy, balsam, flower (*, #)	0.28	1.32	0.00	< 0.05
Nerolidol	0.01	flower, fruit (*, #)	0.00	27.50	0.00	< 0.05
(*E*)-2-Hexen-1-ol	0.1	green, leaf, walnut (*)	0.00	2.76	0.00	< 0.05
farnesene	0.087	citrus, lime, neroli, herbal, myrrh, sweet, bergamot, wood, lavender, green (*, #)	0.00	1.45	0.00	< 0.05
Epoxydihydrolinalool	n. f.	flower, wood (*)	-	-	-	< 0.05
linalool	0.028	lemon, citrus, orange, floral, sweet, woody, blueberry, lavender, flower, green (*, #)	35.99	56.02	52.86	< 0.05

(E)-linalool oxide (pyranoid)	3	Woody, tea-like (#)	0.05	0.18	0.00	< 0.05
Methyl cis-11,14,17-eicosatrienoate	n. f.	n. f.	-	-	-	< 0.05
(Z)-3-hexen-1-yl caproate	n. f.	fruit, prune (*)	-	-	-	< 0.05
beta-calacorene	n. f.	n. f.	-	-	-	< 0.05
3-Phenoxy-1-propanol	n. f.	n. f.	-	-	-	< 0.05
4-Phenyltoluene	n. f.	n. f.	-	-	-	< 0.05
alpha-cadinol	n. f.	herb, wood (*)	-	-	-	< 0.05

Note: OT: odor thresholds, all odor thresholds were obtained from “Compilations of odor thresholds values in air, water and other media”written by (Gemert 2011),or obtained from relevant literature of (Xie, Wang et al. 2023), (Guo, Schwab et al. 2022),(Zhai, Zhang et al. 2022). Odor descriptions are found on the following websites:*:https://www.flavornet.org/flavornet.html; #:https://cosylab.iiitd.edu.in/flavordb/search. “n. f.”, data was not found in the literature.

Table S4. Odor descriptions and ROAV of key differentiating volatiles of finished tea blended with black tea.

Compounds	OT (mg/kg)	Odor Descriptor	ROAV			P
			Fud	AFJG	AFJX	
linalool	0.028	lemon, citrus, orange, floral, sweet, woody, blueberry, lavender, flower, green (*, #)	35.99	44.19	45.75	＜0.05
linalool oxide (pyranoid)	0.19	flower (*)	0.93	1.64	2.53	＜0.05
methyl salicylate	0.04	mint, wintergreen, peppermint (*, #)	10.25	24.55	14.85	＜0.05
Geraniol	0.075	citrus, rose, waxy, floral, sweet, fruity, geranium (*, #)	9.45	21.51	15.41	＜0.05
Benzyl alcohol	0.62	berry, balsamic, rose, floral, walnut, sweet, cherry, phenolic, flower, grapefruit (*, #)	0.92	1.65	0.92	＜0.05
Nerolidol	0.01	flower, fruit (*, #)	0.00	3.10	0.00	＜0.05
dimethyl sulfide	0.01	citrus, cabbage, sulfurous, putrid, wine-like, onion, fatty, floral, earthy, woody, fruity, herbaceous, nutty, meaty, spicy, green (*, #)	77.20	74.53	73.30	＞0.05
Epoxydihydrolinalool	n. f.	flower, wood (*)	-	-	-	＜0.05
dimethyl sulfoxide	n. f.	fatty, mushroom, cheesy, garlic, bitter, oily (*, #)	-	-	-	＜0.05
trans-Anethole	0.015	peppermint, licorice, sweet, anise, balsam, mimosa (*, #)	8.87	9.53	8.40	＞0.05
2-phenylethanol	0.14	lilac, honey, rose flower, floral, spice, bitter, rose dried (*, #)	20.95	20.49	21.56	＞0.05
Methyl hexadecanoate	4	fruit (*)	0.15	0.35	0.21	＜0.05
elaidic acid methyl ester	n. f.	n. f.	-	-	-	＜0.05
methyl linoleate	n. f.	n. f.	-	-	-	＜0.05
Methyl cis-11,14,17-eicosatrienoate	n. f.	n. f.	-	-	-	＜0.05
phytol	0.64	powdery, delicate, waxy, balsam, flower (*, #)	0.28	1.34	0.00	＜0.05

benzyl benzoate	0.341	oil, pineapple, balsamic, herbal, strawberry, oily, faint, sweet, balsam, cherry, almond, herb, cheese (*, #)	0.28	1.05	0.00	＜0.05
styrene	0.004	balsamic, gasoline, floral, sweet, balsam, plastic (*, #)	68.43	68.33	61.30	＞0.05
cis-2-penten-1-ol	0.72	rubber, ethereal, green, fruity, plastic (*, #)	0.00	0.31	0.00	＜0.05
Anethole	n. f.	n. f.	-	-	-	＜0.05
Methyl Pentadecanoate	n. f.	n. f.	-	-	-	＜0.05
Propylcyclopropane	n. f.	n. f.	-	-	-	＜0.05

Note: OT: odor thresholds, all odor thresholds were obtained from "Compilations of odor thresholds values in air, water and other media"written by (Gemert 2011),or obtained from relevant literature of (Xie, Wang et al. 2023), (Guo, Schwab et al. 2022),(Zhai, Zhang et al. 2022). Odor descriptions are found on the following websites:*:https://www.flavornet.org/flavornet.html; #:https://cosylab.iiitd.edu.in/flavordb/search. "n. f.", data was not found in the literature.

Table S5. Odor descriptions and ROAV of key differentiating volatiles of finished tea blended with black tea and fresh leaves blended with black tea.

Compounds	OT (mg/kg)	Odor Descriptor	ROAV				P
			FJG	FJX	AFJX	AFJG	
dimethyl sulfide	0.01	citrus, cabbage, sulfurous, putrid, wine-like, floral, earthy, woody, fruity, herbaceous, green (*, #)	82.60	86.80	73.30	126.10	<0.05
isobutyraldehyde	0.0435	green, malt, fresh, floral, pungent, aldehydic (*, #)	9.15	8.09	12.37	14.00	<0.05
2-methylbutanal	0.0125	cocoa, almond (*)	34.24	31.76	43.12	52.32	<0.05
linalool	0.028	lemon, citrus, orange, floral, sweet, woody, blueberry, lavender, flower, green (*, #)	54.07	50.05	44.19	45.75	<0.05
linalool oxide (pyranoid)	0.19	flower (*)	3.95	5.19	2.53	1.64	<0.05
methyl salicylate	0.04	mint, wintergreen, peppermint (*, #)	16.08	15.63	14.85	24.55	<0.05
Geraniol	0.075	citrus, rose, waxy, floral, sweet, fruity, geranium (*, #)	12.65	13.15	15.41	21.51	<0.05
Benzyl alcohol	0.62	berry, balsamic, rose, floral, walnut, sweet, cherry, phenolic, flower, grapefruit (*, #)	1.25	1.15	0.92	1.65	<0.05
Methyl hexadecanoate	4	fruit (*)	0.20	0.22	0.21	0.35	<0.05
2,4-Di-tert-butylphenol	0.5	n. f.	0.00	0.00	0.00	0.20	<0.05
phytol	0.64	powdery, delicate, waxy, balsam, flower (*, #)	0.00	0.49	0.00	1.34	<0.05
2-hexenal	0.03	leafy, apple, cheesy, vegetable, fat, banana, rancid, fatty,	0.00	0.00	0.00	2.90	<0.05

		sweet, plum, fruity, aldehydic, almond, green (*, #)					
Epoxydihydrolinalool	n. f.	flower, wood (*)	-	-	-	-	<0.05
2-phenylethanol	0.14	lilac, honey, rose flower, floral, spice, bitter, rose dried (*, #)	24.85	20.77	20.49	21.56	>0.05
dimethyl phthalate	n. f.	n. f.	-	-	-	-	<0.05
elaidic acid methyl ester	n. f.	n. f.	-	-	-	-	<0.05
methyl linoleate	n. f.	n. f.	-	-	-	-	<0.05
Methyl cis-11,14,17-eicosatrienoate	n. f.	n. f.	-	-	-	-	<0.05
4-methyldodecane	n. f.	n. f.	-	-	-	-	<0.05
styrene	0.0036	balsamic, gasoline, floral, sweet, balsam, plastic (*, #)	64.75	62.25	55.17	61.33	>0.05
Anethole	n. f.	n. f.	-	-	-	-	<0.05
3-phenylfuran	n. f.	n. f.	-	-	-	-	<0.05
ethyl palmitoleate	n. f.	n. f.	-	-	-	-	<0.05
Methyl Pentadecanoate	n. f.	n. f.	-	-	-	-	<0.05
Propylcyclopropane	n. f.	n. f.	-	-	-	-	<0.05

Note: OT: odor thresholds, all odor thresholds were obtained from “Compilations of odor thresholds values in air, water and other media”written by (Gemert 2011),or obtained from relevant literature of (Xie, Wang et al. 2023), (Guo, Schwab et al. 2022),(Zhai, Zhang et al. 2022). Odor descriptions are found on the following websites:*:https://www.flavornet.org/flavornet.html; #:https://cosylab.iiitd.edu.in/flavordb/search. “n. f.”, data was not found in the literature.

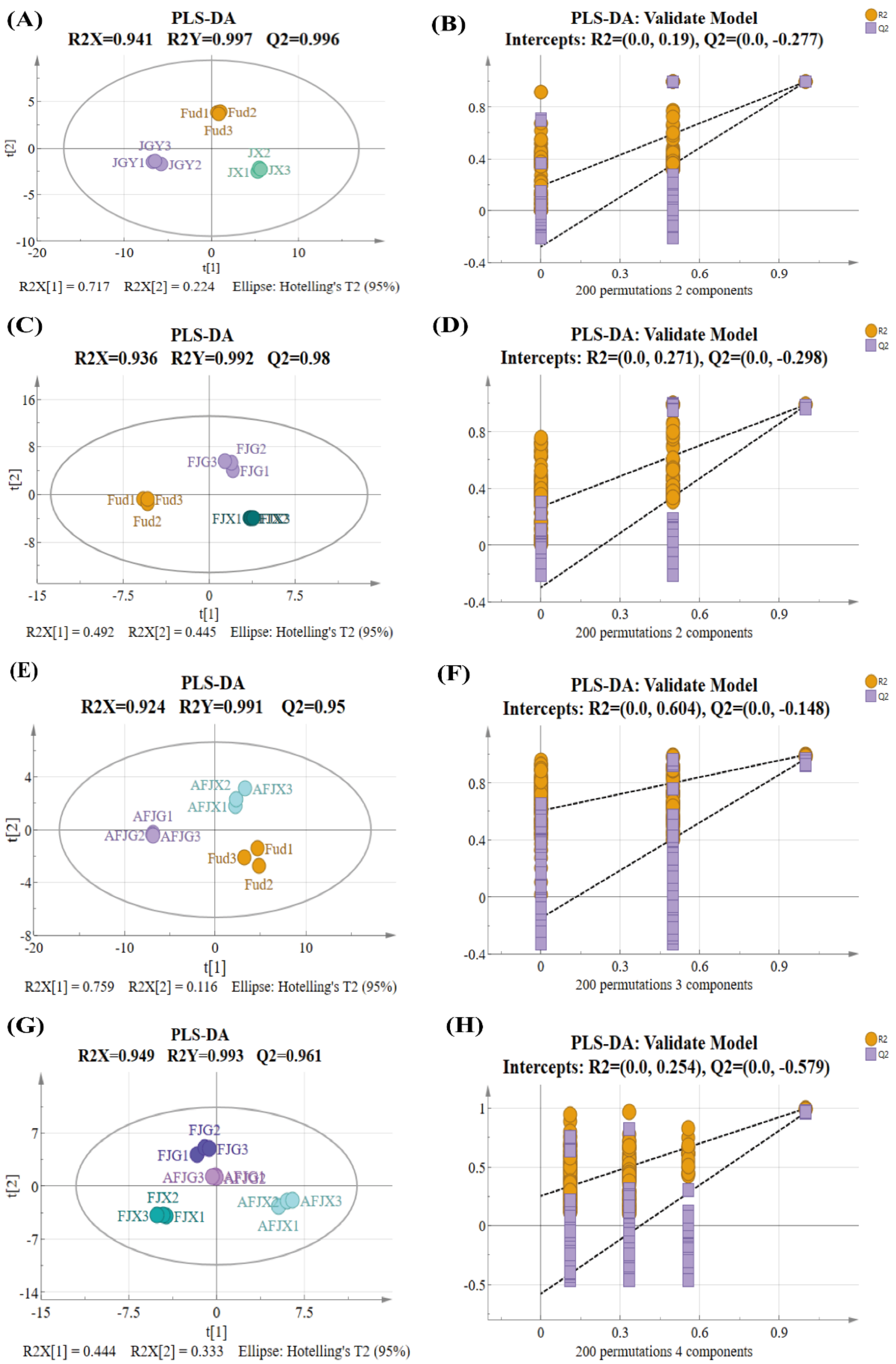

Figure S1. Partial least squares discriminant analysis score chart of maintast components in blending black tea and its raw materials and substitution test.

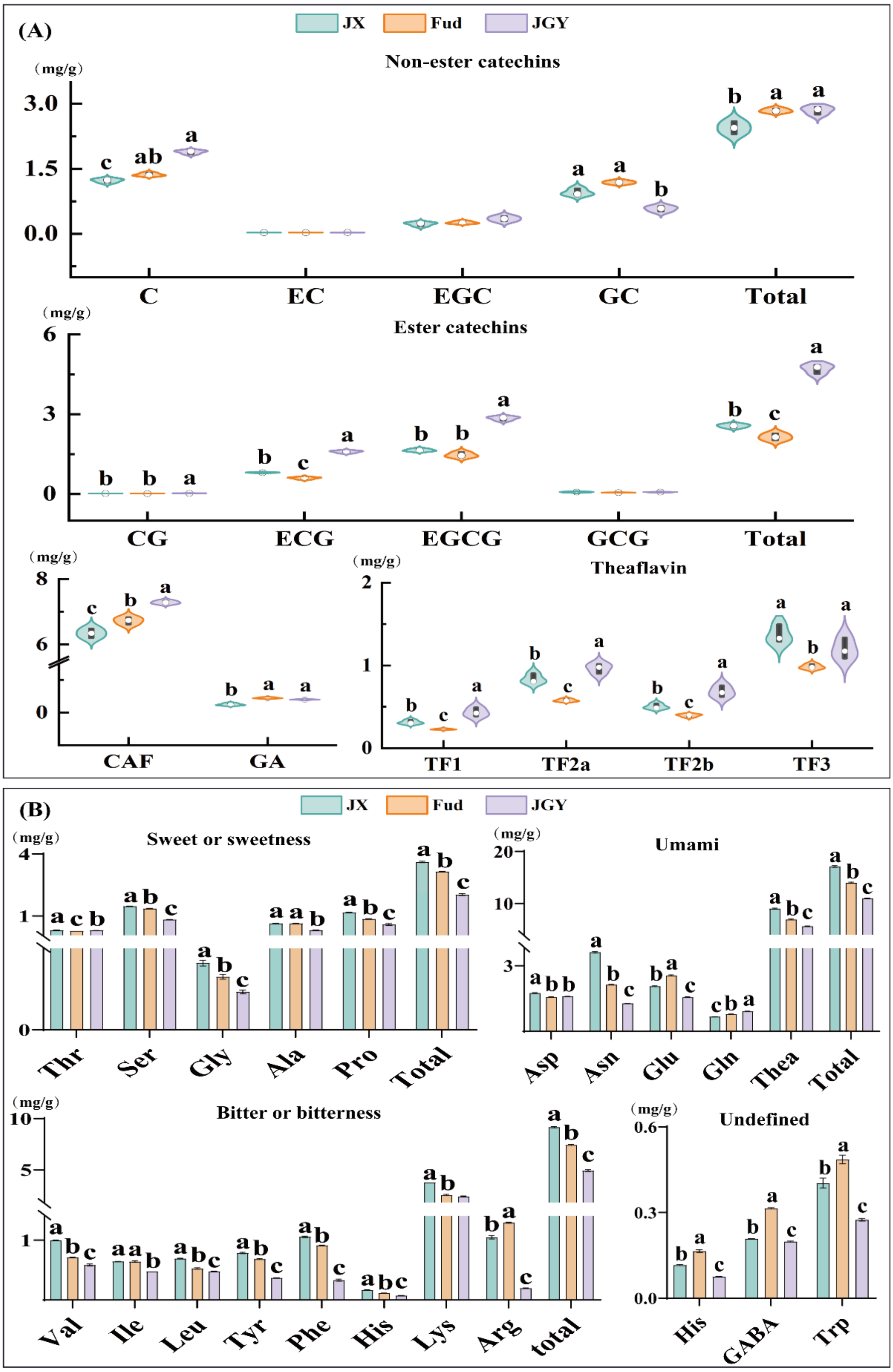

Figure S2. Analysis of the main chemical components (catechins, caffeine, gallic acid, theaflavins and amino acids between of three single varieties of black tea.

Note: different letters indicated statistically significant ($p < 0.05$) between two groups.

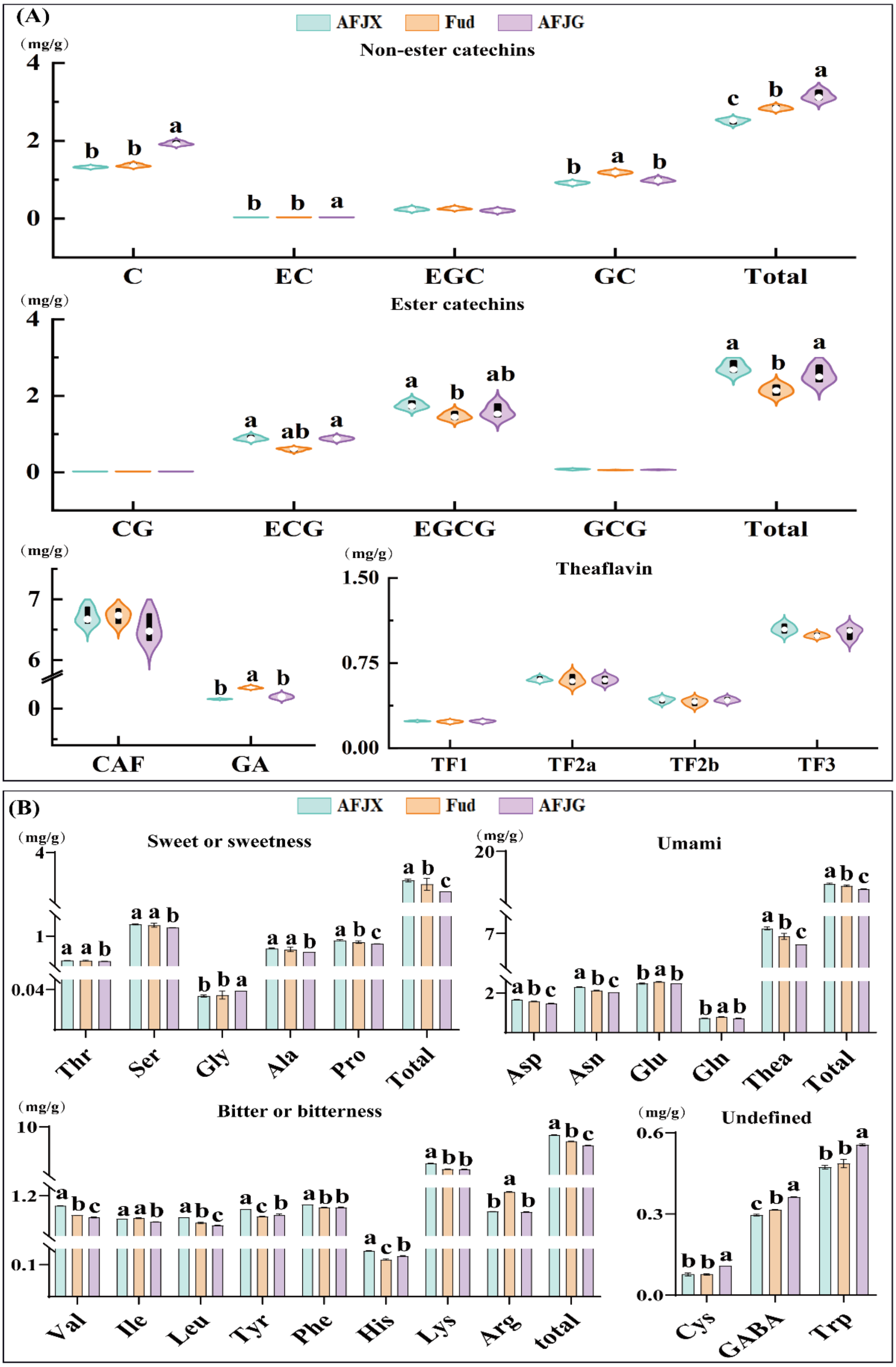

Figure S3. Comparative analysis of the main chemical components (catechins, caffeine, gallic acid, theaflavins and amino acids) of finished tea and Fud black tea. Note: different letters indicated statistically significant (p < 0.05) between two groups.

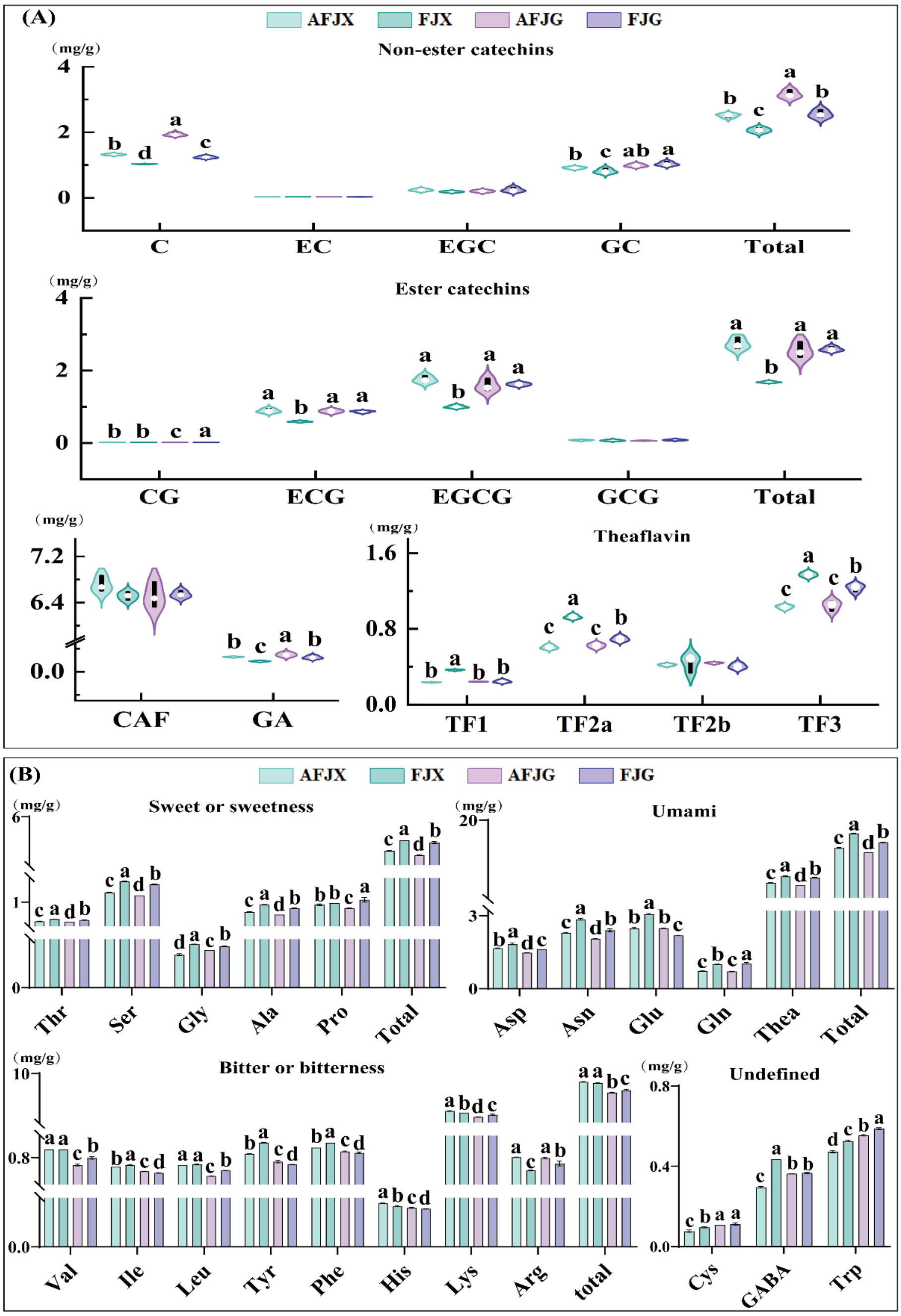

Figure S4. Comparative analysis of the main chemical components (catechins, caffeine, gallic acid, theaflavins and amino acids) of finished tea blended with

black tea and fresh leaves blended with black tea. Note: different letters indicated statistically significant ($p < 0.05$) between two groups.

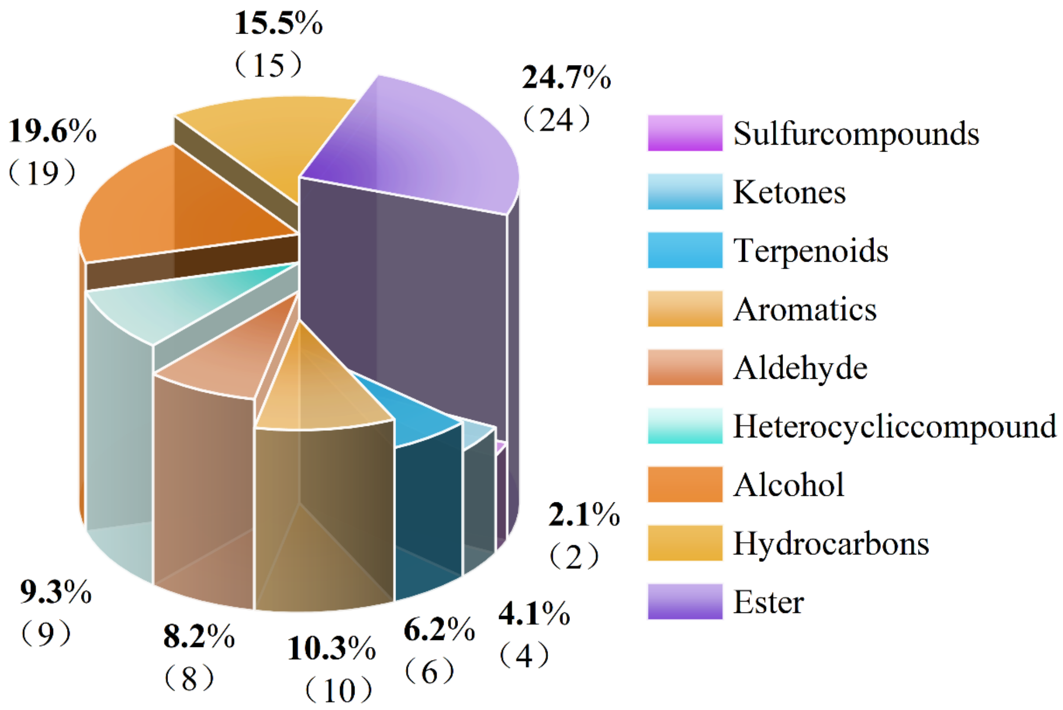

Figure S5. Classification of volatile organic compounds in different blending black tea and its raw materials.

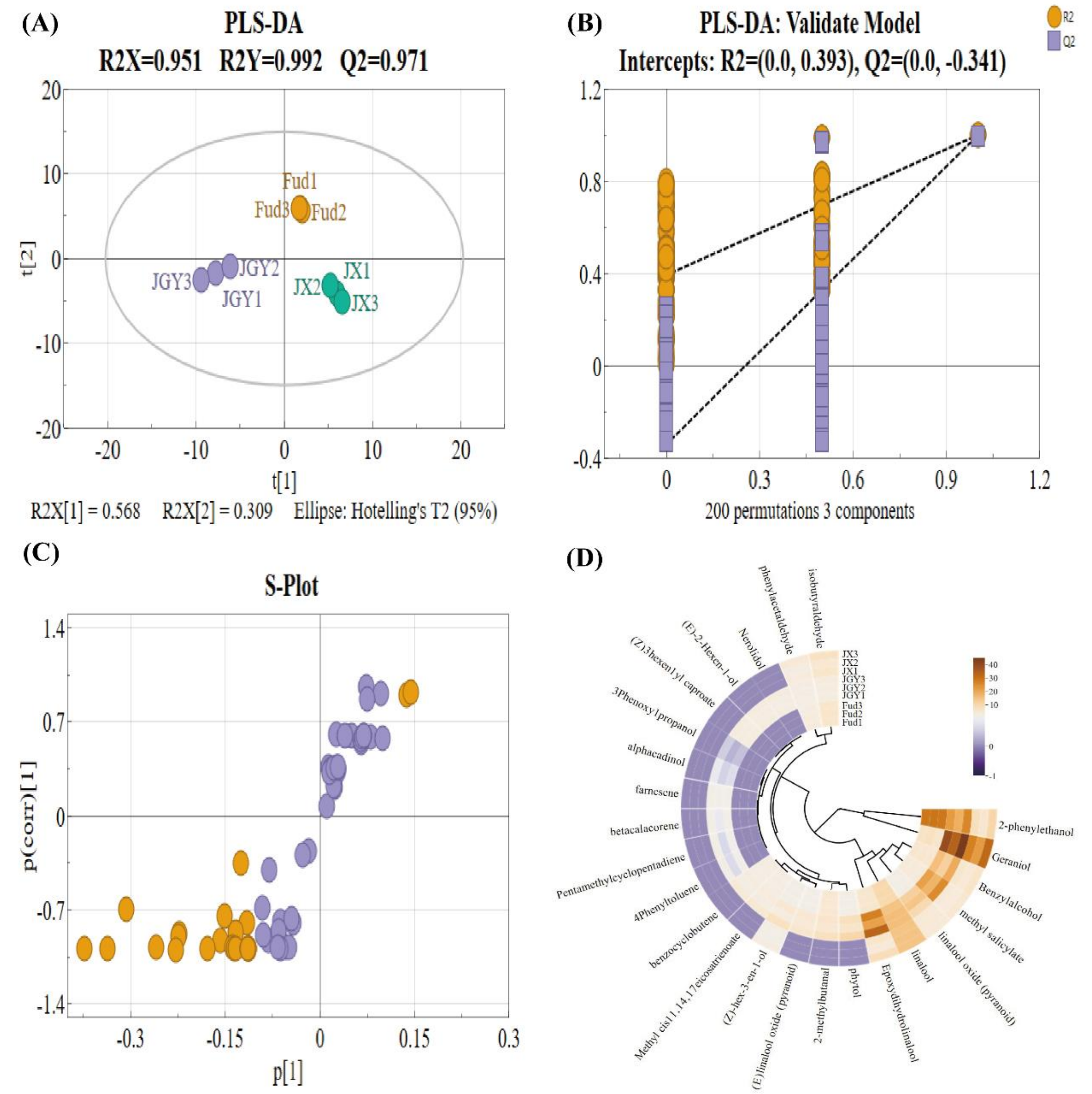

Figure S6. (**A**) Partial least squares discriminant analysis score diagram (PLS-DA) of volatile compounds in three single varieties of black tea. (**B**) Substitution test. (**C**) Differential material screening s-plot. (**D**) Heat map of different key substance content distribution of three single varieties.

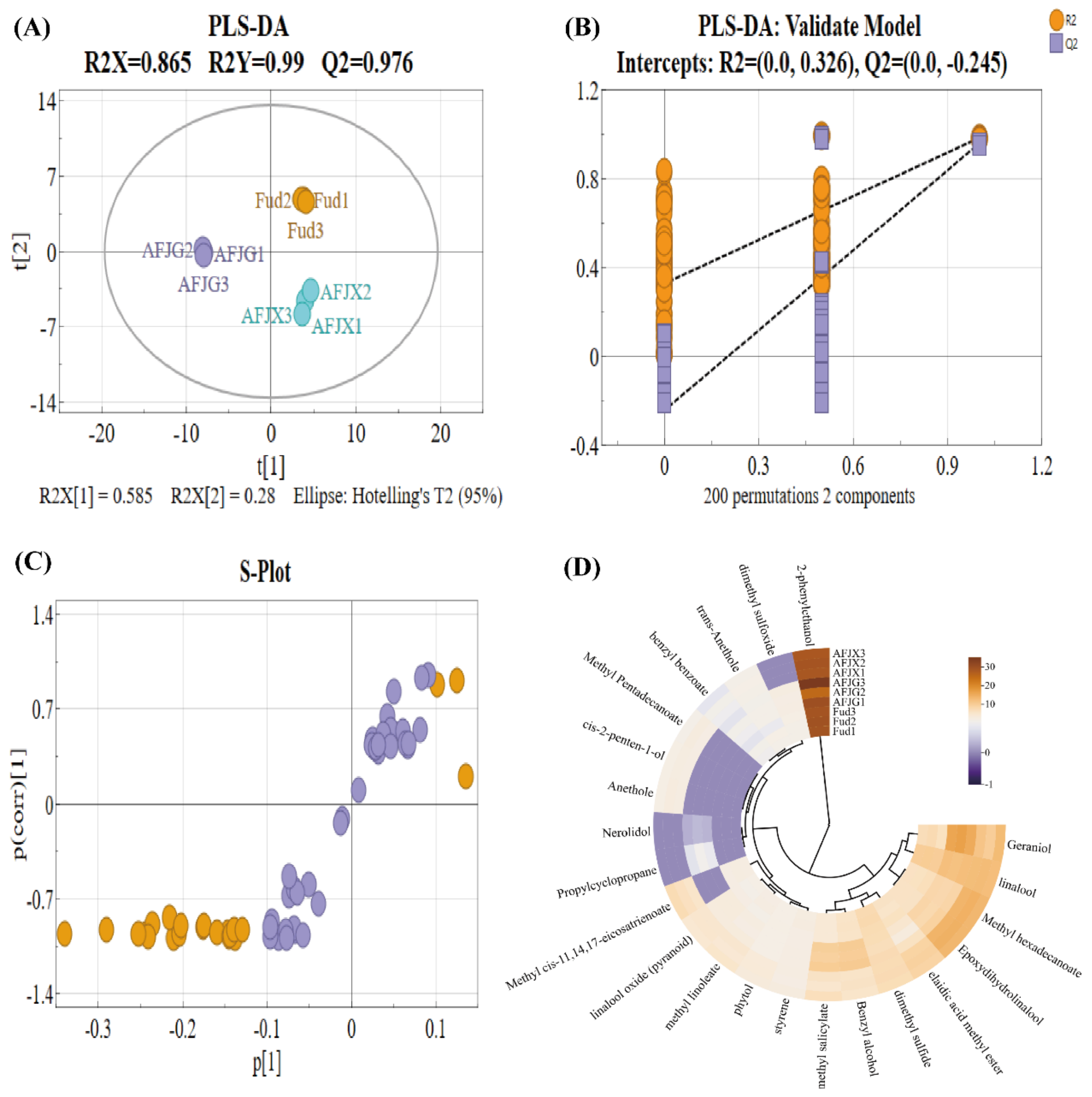

Figure S7. (**A**) Partial least squares discriminant analysis score diagram (PLS-DA) of volatile compounds in finished black tea blended and Fud black tea. (**B**) Substitution test. (**C**) Differential material screening s-plot (**D**) Heat map of different key substance content distribution of finished black tea blended and Fud black tea.

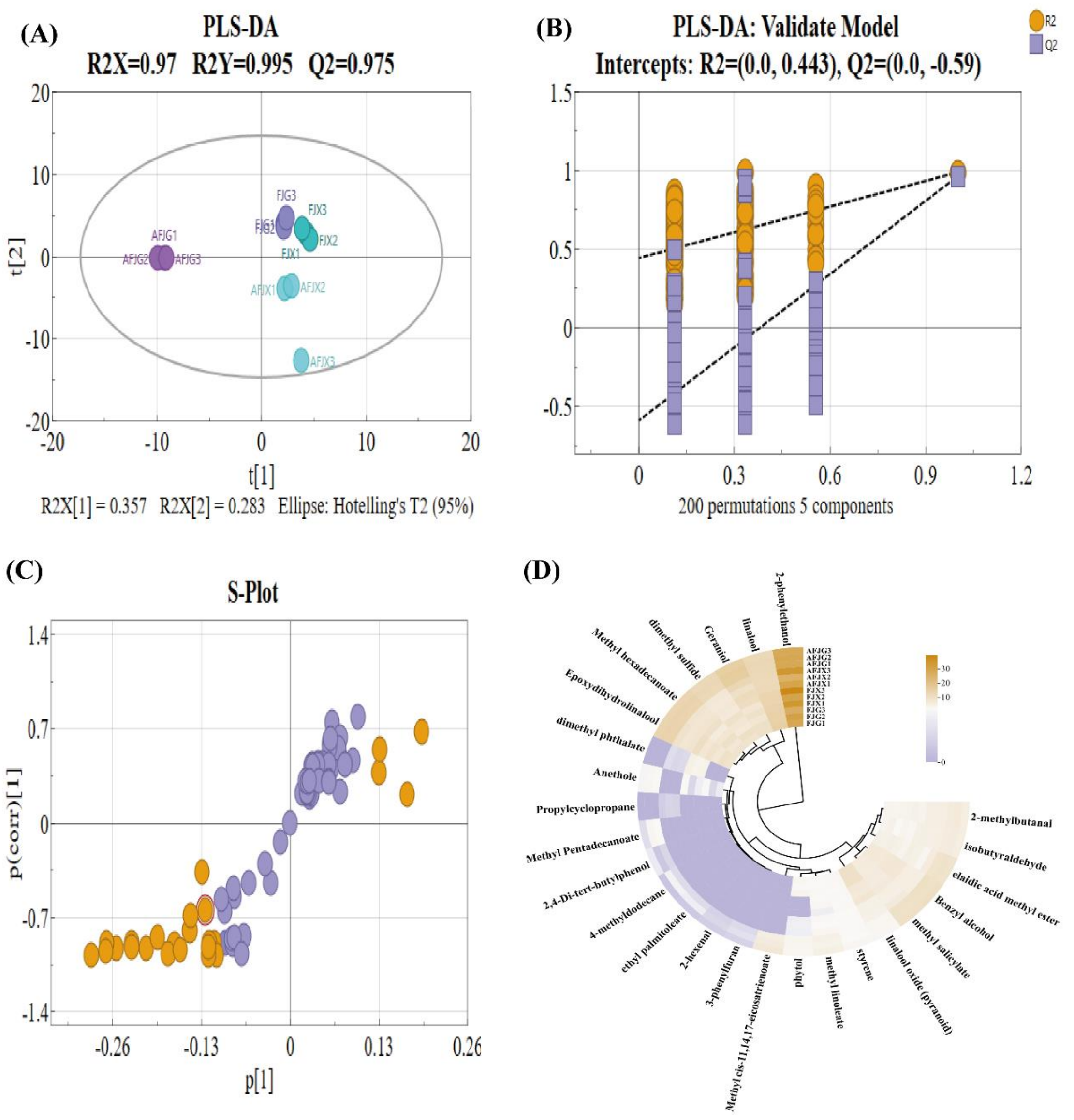

Figure S8. (**A**) Partial least squares discriminant analysis score diagram (PLS-DA) of volatile compounds in finished tea blended with black tea and fresh leaves blended with black tea. (**B**) Substitution test. (**C**) Differential material screening s-plot. (**D**) Heat map of different key substance content distribution of finished tea blended with black tea and fresh leaves blended with black tea.

Gemert, L. J. v. (2011). Compilations of odour thershold values in air, water and other media.

Guo, X., W. Schwab, C.-T. Ho, C. Song and X. Wan (2022). "Characterization of the aroma profiles of oolong tea made from three tea cultivars by both GC–MS and GC-IMS." Food Chemistry 376: 131933.

Xie, J., L. Wang, Y. Deng, H. Yuan, J. Zhu, Y. Jiang and Y. Yang (2023). "Characterization of the key odorants in floral aroma green tea based on GC-E-Nose, GC-IMS, GC-MS and aroma recombination and investigation of the dynamic changes and aroma formation during processing." Food Chemistry 427: 136641.

Zhai, X., L. Zhang, M. Granvogl, C. T. Ho and X. Wan (2022). "Flavor of tea (Camellia sinensis): A review on odorants and analytical techniques." Comprehensive Reviews in Food Science and Food Safety 21(5): 3867-3909.
